# Universal Health Coverage in Francophone Sub-Saharan Africa: Assessment of Global Health Experts' Confidence in Policy Options

**DOI:** 10.9745/GHSP-D-18-00001

**Published:** 2018-06-27

**Authors:** Elisabeth Paul, Fabienne Fecher, Remo Meloni, Wim van Lerberghe

**Affiliations:** a Political Economy and Health Economics, Faculty of Social Sciences, Université de Liège, Liège, Belgium.; b School of Public Health, Université libre de Bruxelles, Brussels, Belgium.; c Independent consultant, Kigali, Rwanda.; d International College of Person-Centred Medicine, Brussels, Belgium.

## Abstract

Many countries rely on standard recipes for accelerating progress toward universal health coverage (UHC). With limited generalizable empirical evidence, expert confidence and consensus plays a major role in shaping country policy choices. This article presents an exploratory attempt conducted between April and September 2016 to measure confidence and consensus among a panel of global health experts in terms of the effectiveness and feasibility of a number of policy options commonly proposed for achieving UHC in low- and middle-income countries, such as fee exemptions for certain groups of people, ring-fenced domestic health budgets, and public-private partnerships. To ensure a relative homogeneity of contexts, we focused on French-speaking sub-Saharan Africa. We initially used the Delphi method to arrive at expert consensus, but since no consensus emerged after 2 rounds, we adjusted our approach to a statistical analysis of the results from our questionnaire by measuring the degree of consensus on each policy option through 100 (signifying total consensus) minus the size of the interquartile range of the individual scores. Seventeen global health experts from various backgrounds, but with at least 20 years' experience in the broad region, participated in the 2 rounds of the study. The results provide an initial “mapping” of the opinions of a group of experts and suggest interesting lessons. For the 18 policy options proposed, consensus emerged only on strengthening the supply of quality primary health care services (judged as being effective with a confidence score of 79 and consensus score of 90), and on fee exemptions for the poorest (judged as being fairly easy to implement with a confidence score of 66 and consensus score of 85). For none of the 18 common policy options was there consensus on both potential effectiveness and feasibility, with very diverging opinions concerning 5 policy options. The lack of confidence and consensus within the panel seems to reflect the lack of consistent evidence on the proposed policy options. This suggests that experts' opinions should be framed within strengthened inclusive and “evidence-informed deliberative processes” where the trade-offs along the 3 dimensions of UHC—extending the population covered against health hazards, expanding the range of services and benefits covered, and reducing out-of-pocket expenditures—can be discussed in a transparent and contextualized setting.

## Abstract (precis)

Even within the fairly homogenous context of francophone Africa, among 18 options presented to experts on how to proceed toward universal health coverage (UHC), consensus was reached on only 1 with respect to effectiveness and another with respect to feasibility. The complexity and challenges of UHC as well as the weak evidence base likely contribute to this uncertainty.

Résumé en français à la fin de l'article. Le texte complet de l'article est aussi disponible en français.

## INTRODUCTION

During the past decade, universal health coverage (UHC) has progressively become global health's number one goal. Progress toward UHC is measured along 3 dimensions: (1) extending the population covered by a mechanism of financial protection against health hazards; (2) expanding the range of services and benefits covered; and (3) reducing out-of-pocket expenditure for the services and benefits that are provided.^1^^–^^3^ However, evidence on the impact of specific UHC design features is scarce and inconclusive,^4^ and it is acknowledged that there is no one-size-fits-all approach to achieving UHC.^2^^,^^5^^–^^8^ Therefore, the choice between the dimension(s) of UHC to be privileged in the first place and the policy options to implement to achieve that implies trade-offs and requires a context-dependent balancing act.^6^ In practice, however, key stakeholders and agencies often tend to sidestep the difficult trade-off decisions by relying on standardized policy heuristics that may override contextualization and negotiation. The influence of global health experts may be very important in this respect.

This article presents exploratory research attempting to measure global health experts' confidence in, and possible consensus on, the effectiveness and feasibility of a number of policy options commonly proposed for achieving UHC in low- and middle-income countries (LMICs). We first present our objectives and the methodological approach we used, and then draw general lessons from our results.

This article attempts to measure global health experts' confidence in and consensus on the effectiveness and feasibility of policy options commonly proposed for achieving universal health coverage.

## METHODS

Our research question emanated from the observation that many countries rely on standard recipes for accelerating progress toward UHC. We aimed to test the following hypothesis: only those policy options that are judged by a majority of experts to be both effective (that is, likely to achieve their intended objective) and feasible (that is, with a reasonable degree of ease in implementation) should be implemented in a vast range of contexts; the others would be used only in favorable, specific contexts. That is why we intended to assess the degree of confidence among a sample of global health experts on the potential effectiveness and feasibility of a set of common policy options, to allow us to move toward UHC in typical African contexts. To do so, we opted for the Delphi method with the hope that we could reach some degree of consensus on at least some policy options that countries could focus on or, alternatively, avoid.

Based on the *World Health Report 2010* on health systems financing,^2^ we identified a number of policy options recommended on the path to UHC and grouped them along the 3 dimensions of the UHC “cube.” We selected the most frequently recommended and implemented in French-speaking sub-Saharan Africa based on our field experience (Table 1). We then performed a selective review of the literature on these policies, in search for systematic evidence when available and non-systematic evidence otherwise.

**TABLE 1.** Common UHC Policy Options Selected for the Delphi Survey

UHC Dimension	Policy Options
Diminish financial barriers to access	B1 Fee exemptions for children and pregnant women B2 Fee exemptions for the poorest B3 Fee exemptions for priority services (e.g., cesarean deliveries, malaria, HIV) B4 Mandatory health insurance with subsidization of the poor B5 Voluntary community-based health insurance B6 Vouchers for the poor

Improve health care funding	F1 Ring-fenced domestic health budgets F2 Innovative financing for health (e.g., sin tax, bonds) F3 Pooling and defragmentation of existing financing mechanisms F4 Reducing inefficiencies and wastage F5 Performance-based aid funding F6 Creation of a global UHC fund

Improve the supply and management of services	S1 Start with a package of essential services for the whole population and progressively expand the package (“universalist” approach) S2 Start with a full package of services for some categories of population or geographic areas, and progressively extend UHC coverage to other types of populations or areas (“sequenced” approach) S3 Strengthen the supply of quality primary health care services S4 Develop public-private partnerships S5 Results- or performance-based payment of providers S6 Separate provider and purchaser functions through creation of autonomous health services or agencies

Abbreviations: B, barriers; F, funding; S, supply; UHC, universal health coverage.

We designed a simple questionnaire comprising explanations about the research's purpose and 2 tables requiring respondents to rate the potential degree of “effectiveness” and “feasibility” of the policy options along a 5-point Likert scale. To limit variability of contexts and ensure coherence, we focused the study on typical contexts of French-speaking sub-Saharan African countries; and to limit ideological biases, we selected a number of global health experts working in various types of organizations (multilateral organizations, bilateral donors, consulting firms or freelance consultants, academia, recipient Ministries of Health) who had experience in this broad region. Experts were selected on an ad hoc basis to represent various profiles, but 2 inclusion criteria were that they had to have (1) at least 20 years' experience in supporting health reforms in LMICs in general, with substantive experience in francophone (Western and/or Central) Africa, and (2) a sufficiently generalist profile in order to be able to assess the effectiveness and feasibility of policies relative to the service provision and the financing dimensions of UHC.

We intended to lead the survey across 2 panels: one composed of 15 experts originating from Europe and Northern America (representing 8 nationalities) (the Northern panel), and one composed of 16 experts from Northern, Western, and Central Africa (representing 11 nationalities) (the Southern panel). We contacted experts individually by email and proposed a discussion of the questionnaire through the use of Skype if they were willing to do so. The experts were blind to each other's scoring and did not interact during the survey. All participants were informed about the study's objectives and methods and consented freely to participate. Anonymity was guaranteed until the last step when we shared results in the aggregate with participating experts. We planned to pursue the Delphi process until a consensus was reached, and to also compare experts' responses with the available evidence on policy options. The 2 rounds of the study were conducted between April and September 2016.

### Adaptations to the Delphi Method

Unexpectedly, our research attempt confronted several issues. First, the participation rate was lower than expected. Of the 31 persons contacted, 23 participated in the first round (13 from the Northern panel, 10 from the Southern panel) and 21 participated in the second round (10 Northern/11 Southern). However, we had to dismiss 4 respondents from the second round who had not participated in the first round, so only 17 experts completed the 2 survey rounds: 9 from Europe (representing 5 nationalities) and 8 from Northern, Western, and Central Africa (representing 8 nationalities). No substantial differences were observed between the 2 panels, so we merged them into a single panel in order to analyze the results. See Table 2 for characteristics of the 17 experts included in our panel.

**TABLE 2.** Characteristics of the Delphi Study Panel of Experts

Expert No.	Country of Origin	Experience in:				
		LMIC MOH Staff or Technical Assistant	Multilateral Organization	Bilateral Agency	Consultancy Firm or Freelance	Academia and/or Civil Society
1	Belgium	X	X		X	X
2	Belgium	X		X		X
3	Belgium				X	X
4	France				X	
5	Germany	X		X	X	
6	Germany	X		X		
7	Germany	X	X	X	X	
8	Italy		X		X	X
9	Netherlands	X	X		X	
10	Burundi	X			X	
11	Democratic Rep. of the Congo	X	X	X	X	
12	Guinea	X	X	X	X	X
13	Côte d'Ivoire	X			X	X
14	Mali	X	X			
15	Morocco	X	X			
16	Senegal	X			X	X
17	Tunisia	X	X			

Abbreviations: LMICs, low- and middle-income countries; MOH, Ministry of Health.

Second, after the first round of the exercise, several experts mentioned the difficulty of granting a score *in abstracto* for each strategy considered in an isolated manner. As happens in the literature,^2^^,^^6^^,^^7^ they commented on the need to contextualize, to consider how strategies interact with each other, and to differentiate between strategies that may be effective at the micro level but might not be easily scaled up at the country level (the “micro-macro” paradox^9^). For instance, while there is strong evidence that community-based health insurance (CBHI) schemes improve service utilization and protect members financially by reducing their out-of-pocket expenditure, and that CBHI improves resource mobilization,^10^ both theory and evidence suggest that a CBHI model, relying only on voluntary, small-scale schemes and small pools with little or no subsidization of poor and vulnerable groups, can play only a very limited role in helping countries move toward UHC.^11^

Therefore, per the participants' request, we converted the initial questionnaire to include 2 sets of 2 questions for the second round. The first set of questions focused on confidence in the effectiveness of each policy option. These questions were broken down into 2 items and thus scored twice, once for its potential effectiveness as a stand-alone intervention, and once for its potential as part of a UHC reform package. The second set of questions focused on confidence in feasibility of each policy option. These questions were scored once for operational and technical feasibility, and once for political feasibility. Yet when asked to differentiate between stand-alone and part-of-package effectiveness, and between technical and political feasibility, the differences in scores were negligible. Consequently, we merged these when analyzing the results.

Third, during the first round, experts gave a “moderate” response to the majority of policy options, thus not clearly positioning themselves in favor for or against proposed policy options. To allow for more clear-cut answers, we converted the Likert scale's results into a nominal scale from 0 to 100, and asked the second round's respondents to use the nominal scale to position themselves in the range between their previous response and the average response. However, during the second round, most policy options kept scoring in the “moderately effective” range, and no consensus emerged as we hoped would be possible.

Therefore, we changed our analytical approach: since no consensus seemed to emerge through the use of the Delphi method, instead, we statistically analyzed the results from our 17 questionnaires. We measured the degree of consensus on each policy option through 100 minus the size of the interquartile range (IQR) of the individual scores, with 100 signifying total consensus.^12^ This indicates an important methodological limitation of our study, due to a lack of representativeness of the expert sample. Our panel was initially designed in a purposive way, fit for the initial qualitative approach chosen, in order to represent a wide range of experiences; however, it is not representative of all global health experts. Therefore, our quantitative results must be interpreted with caution. While providing an indication of tendencies, they certainly should not to be thought of as being generalizable. Other limitations of our study deal with the focus on French-speaking Africa, which is quite homogeneous in some respects—notably the epidemiological profile and main features of health systems and policies—but which also gathers, under the same umbrella, very different contexts, notably in terms of wealth and political regimes. Experts' opinions might also have been biased by their personal experience, be it positive or negative, in specific countries.

## FINDINGS

### Statistical Analysis of Delphi Survey Responses

Regarding potential effectiveness, only 8 policy options received a consensus rating of 80% or above, but 7 of them were in the “moderately effective” range (confidence scores between 48% and 60%), indicating “soft consensus” (Table 3). Only the policy option **“strengthen the supply of quality primary health care services”** was consensually judged as being clearly effective by our panel (confidence score of 79 with a consensus rating of 90).

**TABLE 3.** Average Scores of Delphi Study Participants for Confidence in and Degree of Consensus^a^ on Effectiveness and Feasibility of Common UHC Policy Options, Classified by Increasing Confidence in Effectiveness

UHC Dimension Code	Policy Option	Effectiveness		Feasibility	
		Confidence	Consensus	Confidence	Consensus
B5	Community-based health insurance	40	75	49	60
F5	Performance-based aid funding	43	72	48	72.5
S5	Performance-based payment of providers	48	67.5	49	80
F1	Ring-fenced budgets	48	80	56	73.75
S2	Expansion of population covered	49	80	54	70
F2	Innovative financing	51	80	59	65
S4	Public-private partnerships	54	80	58	72.5
B2	Fee exemption for poorest	55	80	66	85
B6	Vouchers	56	80	63	75
F4	Reduction of inefficiencies	59	75	60	75
B1	Fee exemption for children and pregnant women	60	80	64	77.5
F3	Pooling of schemes	60	75	57	65
S6	Purchaser-provider split	60	75	60	68.75
B3	Fee exemption for specific services	61	75	65	77.5
F6	Global fund for UHC	64	75	67	67.5
S1	Expansion of package of services	64	76.25	68	75
B4	Mandatory health insurance	67	75	68	70
S3	Strengthen supply of quality primary health care	79	90	78	73.75

Abbreviations: B, barriers; F, funding; S, supply; UHC, universal health coverage.

a Degree of consensus measured through 100 (total consensus) minus the interquartile range of individual scores.

Strengthening the supply of primary health care services was the only policy option that the panel of experts agreed was effective for achieving UHC.

Regarding feasibility, the degree of consensus was even lower. Only 2 policy options received a consensus rating of 80 or above (performance-based payment of providers and fee exemption for the poorest), but one of them was judged moderately feasible (with a confidence score of 49), so that only the policy option **“fee exemption for the poorest”** was consensually judged as being fairly easy to implement by our panel (confidence score for feasibility of 66, consensus rating of 85). On the other hand, for 5 policy options, the consensus rating was below 70, indicating very diverging opinions among the experts.

### Review of Key Evidence on Policy Options

To deepen our analysis, we attempted to compare responses from our panel of experts with the available evidence (not especially related to French-speaking sub-Saharan Africa), in order to assess whether or not each corresponded with the other. We therefore opted to search for systematic evidence with regard to each of the 18 proposed policy options and completed the literature review with non-systematic evidence when needed.

Regarding the path toward UHC, overall, the existing literature concludes that the effect of UHC schemes on access, financial protection, and health status varies across contexts, UHC scheme design, and the implementation processes. In most countries and regions, a number of UHC schemes coexist although they demonstrate heterogeneity in terms of design and organization. Evidence on the impact of specific UHC design features on their intended outcomes is scarce and inconclusive, so that several pathways may be appropriate.^4^^–^^7^^,^^13^^,^^14^ Yet, there are indications that a progressive expansion of a package of essential services for the whole population (sometimes called progressive universalism) is preferable to sequential extension of the share of the population covered with a full package of services.^14^^–^^17^ However, to our knowledge, no systematic evidence is available to back this opinion. The literature is also consensual on the fact that no country has attained UHC by relying mainly on voluntary contributions to insurance schemes^18^^,^^19^ and progress toward UHC requires compulsion and cross-subsidization.^6^^,^^19^^,^^20^

Progressive expansion of a package of essential services for the whole population may be preferable to sequential extension of the share of the population covered with a full package, but no systematic evidence is available.

Regarding specific policy options, we found that the lack of confidence and consensus among our experts, with large inter-expert variability (ranging from 0 to 100 in the case of fee exemption for the poorest) and many policy options lying in the “woolly” consensus area, often matches the **lack of consistent evidence on the proposed policy options**. Indeed, the literature is often scanty, inconclusive, and inconsistent, or may challenge the implementation of the proposed policy options. This is the case for:

- *Fee exemptions*: They are shown to entail many implementation challenges while robust evidence quantifying their impact remains scant.^21^^–^^26^
- *Ring-fenced domestic budgets for health*: The effectiveness of earmarking taxes or revenue for health appears to be mixed in terms of increasing overall funding, improving its stability, or increasing expenditure for the target program.^5^^,^^27^^–^^29^
- *Innovative financing mechanisms*: These fall in various categories, but their potential impact on efficiency and equity depend on each country context.^30^^,^^31^
- *Mandatory health insurance*: A systematic review of the impact of state-subsidized or social health insurance schemes found no strong evidence of any impact on utilization, protection from financial risk, or health status.^32^ Moreover, the real issue is more about the feasibility of the strategy, in terms of ensuring enrollment in a health insurance mechanism, because the willingness to pay for health insurance in LMICs is low,^33^ and even subsidized schemes for the non-poor informal sector face low participation and retention issues.^32^
- *Performance-based financing*: There is no consistent evidence of the effectiveness, efficiency, and equity of the approach, and there are indications of possible perverse effects on health care providers' behavior and weakening of health systems.^34^^–^^41^
- *Public-private partnerships*: Despite the scale and significance of the phenomenon, there is relatively limited conceptualization and in-depth empirical investigation of such partnerships. Evidence about their effect on clinical quality, coverage, equity, and cost-effectiveness is inadequate, and other challenges concern scalability and scope, indicating the limitations of such interventions as a basis for universal health coverage, though interventions can address focused problems on a restricted scale.^42^^,^^43^
- *Purchaser-provider splits:* No systematic review or coherent literature has been found on this matter, probably due to the extreme diversity in approaches.
- *Performance-linked aid*: Its alleged advantages are flawed theoretically and poorly backed by empirical research.^44^^–^^48^

Despite the scale and significance of public-private partnerships, there is relatively limited conceptualization and in-depth empirical evidence of such partnerships.

On the whole, the quality of the evidence is low and/or non-systematic. The panel's relative confidence in mandatory health insurance, purchaser-provider split, and the creation of a global fund for UHC contrasts with the lack of documented evidence for these options.

On the other hand, the literature does provide evidence that several policy options, with regard to which our experts had mixed opinions, **can contribute positively to UHC**. This is the case for:

- *Community-based health insurance*: There is systematic but weak evidence of a moderate effect with improved service utilization and financial protection by reducing out-of-pocket spending; yet, schemes serve only a limited section of the population.^10^^,^^49^
- *Vouchers*: A systematic review of the impact of vouchers on the use and quality of health care in developing countries found modest evidence that vouchers effectively target specific populations, but there is insufficient evidence to determine whether vouchers deliver health care efficiently. There is robust evidence that vouchers increase utilization, and modest evidence that vouchers improve quality,^50^ in particular for reproductive health services. Another systematic review concluded that all identified evaluations reported some positive findings, indicating that voucher programs increase the utilization of reproductive services, improve quality of care, and improve population health outcomes.^51^ A related strategy is the use of *conditional cash transfers*: Overall the evidence suggests that they are effective in increasing the use of preventive services and sometimes improving health status.^52^ A recent systematic review focusing on sub-Saharan Africa found that cash transfers can be effective in tackling structural determinants of health such as financial poverty, education, household resilience, child labor, social capital, and social cohesion. The review further found that cash transfers modify intermediate determinants such as nutrition, dietary diversity, child deprivation, sexual risk behaviors, teen pregnancy, and early marriage. There is moderate evidence from the review that cash transfers impact health and quality-of-life outcomes.^53^
- *Pooling/defragmentation:* There is anecdotal evidence of a positive effect, even if experience suggests that once established, different pools are politically difficult to integrate or harmonize because integration involves the redistribution of resources across organized interest groups.^15^^,^^17^^,^^19^^,^^54^^–^^56^
- *Reduction of inefficiencies:* There is non-systematic evidence of a positive effect,^57^ and systematic evidence of cost savings and efficiency gains in HIV services in LMICs.^58^
- *Strengthening primary health care*: The experts on our panel were quite confident with regard to the potential of this policy option, which is also supported by existing evidence (mainly non-systematic evidence,^5^^,^^55^^,^^59^^,^^60^ but also by systematic evidence in high-income countries,^61^^,^^62^ and limited systematic evidence in LMICs).^63^^,^^64^ For instance, a systematic review found that although a majority of primary care programs had multiple components—thus making it difficult to attribute effects to the primary care component alone given this integration and the variable quality of the available research—primary care-focused health initiatives in LMICs have improved access to health care, including among the poor, at reasonably low cost. There is also evidence that primary care programs have reduced child mortality and, in some cases, wealth-based disparities in mortality.^64^

## DISCUSSION

First, note that since our panel of experts was built on an ad hoc basis in order to involve a wide range of experienced, generalist global health experts, it cannot be characterized as an epistemic community, that is “a network of professionals with recognized expertise and competence in a particular domain and an authoritative claim to policy-relevant knowledge within that domain or issue-area.”^65^ Indeed, if the inclusion criteria were aimed to guarantee shared knowledge base about UHC and possibly shared principled beliefs, their actual heterogeneity in terms of personal and professional experience—which was desired in the first place—might have led to different causal beliefs and possibly different interests or ideological values. This might have led to the impossibility of reaching a consensus through the Delphi method.

Our study provides an initial “mapping” of the opinions of a group of experts, which offers interesting lessons. It shows that among a panel of 17 experienced global health experts coming from varied countries and backgrounds, **none of the 18 common policy options recommended on the path toward UHC received sufficient consensus regarding both its potential effectiveness and its feasibility**. This lack of clarity and consensus of opinions regarding UHC policy options in a relatively homogeneous region like French-speaking sub-Saharan Africa is itself an important piece of information. According to our basic premise, this suggests that we should be cautious when it comes to implementing these policy options and that they should be implemented only in favorable contexts, following a careful analysis and trade-off process. But this is not the case in practice, since we observe the concomitant implementation of many of the proposed options in most sub-Saharan African countries and the trend of some donor agencies to “sell” preferred policy options whatever the context. The most striking example is probably that of performance- or results-based financing: This is the one policy option that received the least consensus among our panel of experts, which contrasts with its rapid donor-funded expansion in LMICs. For example, since its inception in 2007, the Health Results Innovation Trust Fund managed by the World Bank has committed $385.6 million for 35 results-based financing programs in 29 countries, with the bulk of disbursements taking place in the past 3 years (https://www.rbfhealth.org/projects). This was done despite the fact that in 2014, the Independent Evaluation Group of the World Bank raised a flag by pointing out that the Bank had promoted results-based financing with insufficient empirical support on the soundness of the approach, even proclaiming that “… decisions were made to scale up regardless of weak, inconclusive, or incomplete pilot results.”^66^

None of the 18 common UHC policy options received sufficient consensus on both potential effectiveness and feasibility.

Our findings suggest UHC policy options should be implemented only in favorable contexts, following a careful analysis and trade-off process.

When comparing our results with findings in the literature, we were struck by the lack of consistent, systematic evidence on the effectiveness of most policy options. Most worrying, even supposedly systematic evidence may not be entirely trusted when applied to complex issues. For instance, Coarasa et al. (2017) examined 2 systematic reviews of the literature on the quality of private-sector primary care in LMICs, published in the same journal within a year and reaching conflicting conclusions. They found that weaknesses in the underlying evidence, rather than the rigor of the reviews themselves, led to reasonable disagreements, and therefore **called for high-quality empirical evidence on reforms aimed at reaching UHC**.^67^ Given the broadness of the UHC objective and the complexity and difficulty of implementing policy reforms—especially in low-resource environments such as in French-speaking sub-Saharan Africa—and given the lack of robust evidence on possible policy options, it may therefore be considered very rational for experts to differ widely in terms of what they might recommend. This stresses the **importance of contextualized policy advice**.

We were also struck by the weak differentiation on the part of the experts between policy options that are isolated vs. part-of-package effectiveness on the one hand, and between technical vs. political feasibility on the other hand (as explained above, the differences in scores were negligible, so that we merged these 2 sets of questions when analyzing the results). We hypothesize that there is a disjunction between experts' professional rationality (they are intellectually convinced of the need to contextualize and to differentiate between effectiveness and feasibility in complex systems) and a rather heuristic appraisal when asked to judge a strategy. This has long been recognized,^68^^,^^69^ and shows the difficulty of formulating objective recommendations in a complex system. It might also raise doubts as to how vulnerable to biases knowledgeable and experienced experts may be when making policy recommendations.

On this issue, Cairney and Oliver (2017) identify 2 important dilemmas for scientists and researchers—that we can extrapolate to advisers—contributing to an “evidence-policy gap.” First, effective actors combine evidence with manipulative emotional appeals to influence the policy agenda. Second, when adapting to multilevel policymaking, experts should not necessarily prioritize “evidence-based” policymaking above other governance principles such as the “coproduction” process of policy between local public bodies, interest groups, and service users, which may be based primarily on values. They conclude that successful engagement in evidence-based policymaking requires pragmatism, combining scientific evidence with governance principles, and persuasion to translate complex evidence into simple stories. To maximize the use of scientific evidence with regard to public health policy, experts should recognize the tendency of policy makers to base judgements on their beliefs and shortcuts based on their emotions and familiarity with information; be prepared to engage in long-term strategies to be able to influence policy; and, in both cases, decide how far they are willing to go to persuade policy makers to act and secure a hierarchy of evidence underpinning policy. These are value-driven and political, not just “evidence-based,” choices.^70^

Finally, we wondered why international organizations often continue to promote a number of strategies (“donor fads”), the effectiveness of which lack consistent evidence. Wane (2004) establishes both theoretically and empirically that the quality of aid is endogenous to the incentive system that prevails in the aid agency (career concerns) and the capacity and accountability of the recipient country to gauge the quality of aid. He shows that a mix of factors (recipient governments' incentives to accept projects if they bring personal benefits, a donor agency culture of “pushing money,” low-capacity and/or low-accountability recipient governments) leads to recipient governments accepting poorly designed projects to the detriment of their population.^71^

## CONCLUSIONS

Despite the limitations pointed out above, and even when limiting the focus to the relatively homogeneous contexts of French-speaking sub-Saharan Africa, **this exploratory study shows that global health experts' opinions on policy options with regard to achieving UHC diverge a great deal, they are often only moderately supportive or not in support of the standard UHC recipes prevalent in today's global health and development rhetoric, and that most policy options are not backed by systematic and coherent evidence**. This conveys implications for the development community. Development agencies are understandably tempted to promote “magic bullet” approaches to UHC. Notably due to a culture of “pushing money” and low-capacity recipient governments,^71^ incentives on both the donor and the recipient side favor simple, easily budgeted, single-instrument or isolated solutions, which can then rapidly proliferate, even with little evidence. This may do more harm than good.^20^ Expert opinion has a large role to play in justifying policy choices, but it would be dangerous to take their opinion for granted—all the more because, despite a discourse of evidence-based decision making, development actors may have opinions based primarily on personal values and may combine evidence with manipulative emotional appeals to influence the policy agenda.^70^ This makes expert policy advice value-driven and political, not just “evidence-based.”

Therefore, there is clearly a need for a better understanding of the interactions between the multiple stakeholders—including not only the experts but also the domestic actors—their agendas, and their hierarchy of values. While progress toward UHC has taken various forms around the globe, a common feature emerging from experience is that adopting UHC is primarily a political rather than a technical issue.^72^^,^^73^ Hence, the **need to strengthen inclusive and “evidence-informed deliberative processes” enabling stakeholders to discuss the trade-offs along the 3 dimensions of UHC in a transparent way, to make the set of values and decision criteria used more explicit, and to take decisions in a coherent and contextualized way**.^74^^–^^77^ This might also promote more actively the respect for domestic political ownership.

## Supplementary Material

18-00001-Paul-FrenchSupplement.pdf

## Notes

**En français**

**La couverture santé universelle en Afrique francophone au Sud du Sahara : Évaluation de la confiance d'un panel d'experts en santé mondiale quant à une série d'options stratégiques**

Même dans le contexte assez homogène de l'Afrique francophone, parmi 18 options stratégiques présentées à des experts permettant de progresser vers la couverture santé universelle (CSU), un consensus n'a été atteint que pour une option quant à son efficacité, et pour une autre quant à sa faisabilité. La complexité et les défis liés à la CSU ainsi que la faiblesse de la base de données probantes contribuent probablement à cette incertitude.

**RÉSUMÉ**

De nombreux pays s'appuient sur des recettes standards pour accélérer les progrès vers la couverture santé universelle (CSU). Les données empiriques généralisables étant limitées, la confiance des experts et leur consensus jouent un rôle majeur dans les choix politiques des pays. Cet article présente une tentative exploratoire menée entre avril et septembre 2016 pour mesurer la confiance et le consensus parmi un panel d'experts en santé mondiale quant à l'efficacité et la faisabilité d'un certain nombre d'options stratégiques communément proposées pour atteindre la CSU dans les pays à revenus faibles et intermédiaires, telles que les exemptions de frais pour certains groupes de personnes, les recettes affectées pour la santé et les partenariats public-privé. Pour assurer une relative homogénéité des contextes, nous nous sommes concentrés sur l'Afrique subsaharienne francophone. Nous comptions d'abord utiliser la méthode Delphi pour arriver à un consensus entre les experts, mais comme aucun consensus ne s'est profilé après deux cycles, nous avons ajusté notre approche pour passer à une analyse statistique des résultats de notre questionnaire. Nous avons mesuré le degré de consensus sur chaque option stratégique à travers 100 (signifiant le consensus total) moins l'intervalle interquartile des scores individuels attribués aux options stratégiques. Dix-sept experts en santé mondiale de divers horizons, mais ayant au moins 20 ans d'expérience dans la vaste région, ont participé aux deux cycles de l'étude. Les résultats fournissent une première « cartographie » des opinions d'un groupe d'experts et suggèrent des leçons intéressantes. Pour les 18 options stratégiques proposées, un consensus s'est seulement dégagé en faveur du renforcement de l'offre de services de soins de santé primaires de qualité (jugé efficace avec un score de confiance de 79 et un score de consensus de 90) et quant à la faisabilité des exemptions de frais pour les plus pauvres (jugées assez faciles à mettre en œuvre avec un score de confiance de 66 et un score de consensus de 85). Aucune des 18 options stratégiques communément rencontrées n'a fait l'objet d'un consensus à la fois quant à son efficacité potentielle et à sa faisabilité. Des opinions très divergentes ont été observées pour cinq options stratégiques. Le manque de confiance et de consensus au sein du groupe d'experts semble refléter le manque de preuves cohérentes sur les options proposées. Cela suggère que les opinions des experts devraient être formulées dans le cadre de processus délibératifs renforcés, inclusifs et fondés sur des données probantes, où les compromis quant aux trois dimensions de la CSU – étendre la population couverte contre les risques de santé, élargir la gamme des services et des avantages couverts, réduire les dépenses directes de santé – peuvent être discutés dans un cadre transparent et contextualisé.

## Notes

**Peer Reviewed**

**Funding:** The contribution of EP and FF was supported by the “Effi-Santé” project funded through the ARC grant for Concerted Research Actions, financed by the French Community of Belgium (Wallonia-Brussels Federation).

**First Published Online:** May 29, 2018

## References

[1] World Health Organization (WHO). The World Health Report 2008: Primary Health Care, Now More than Ever. Geneva: WHO; 2008. http://www.who.int/whr/2008/en/. Accessed November 13, 2017.

[2] World Health Organization (WHO). The World Health Report 2010: Health Systems Financing, The Path to Universal Coverage. Geneva: WHO; 2010. http://www.who.int/whr/2010/en/. Accessed November 13, 2017. 10.2471/BLT.10.078741 PMC2878164 20539847

[3] Busse R Schreyogg J Gericke C. Analyzing Changes in Health Financing Arrangements in High-Income Countries: A Comprehensive Framework Approach. HNP discussion paper. Washington, DC: World Bank; 2007. http://documents.worldbank.org/curated/en/162311468141575894/Analyzing-changes-in-health-financing-arrangements-in-high-income-countries-a-comprehensive-framework-approach. Accessed November 13, 2017.

[4] Giedion U Alfonso EA Diaz Y. The Impact of Universal Coverage Schemes in the Developing World: A Review of the Existing Evidence. Universal Health Coverage (UNICO) Studies Series No. 25. Washington, DC: World Bank; 2013. http://documents.worldbank.org/curated/en/349621468158382497/The-impact-of-universal-coverage-schemes-in-the-developing-world-a-review-of-the-existing-evidence. Accessed November 13, 2017.

[5] Maeda A Cashin C Harris J Ikegami N Reich MR. Universal Health Coverage for Inclusive and Sustainable Development: A Synthesis of 11 Country Case Studies. Washington, DC: World Bank Group; 2014. http://documents.worldbank.org/curated/en/575211468278746561/Universal-health-coverage-for-inclusive-and-sustainable-development-a-synthesis-of-11-country-case-studies. Accessed November 13, 2017.

[6] Reich MR Harris J Ikegami N. Moving towards universal health coverage: lessons from 11 country studies. Lancet. 2016;387(10020):811–816. 10.1016/S0140-6736(15)60002-2. 26299185

[7] Cotlear D Nagpal S Smith O Tandon A Cortez R. Going Universal: How 24 Developing Countries Are Implementing Universal Health Coverage Reforms from the Bottom Up. Washington, DC: World Bank; 2015. http://documents.worldbank.org/curated/en/936881467992465464/pdf/99455-PUB-Box393200B-OUO-9-PUBDATE-9-28-15-DOI-10-1596-978-1-4648-0610-0-EPI-210610.pdf. Accessed November 13, 2017.

[8] World Bank. UHC in Africa: A Framework for Action. Washington, DC: World Bank; 2016. http://documents.worldbank.org/curated/en/735071472096342073/Main-report. Accessed November 13, 2017.

[9] Mosley P. Aid-effectiveness: the micro-macro paradox. IDS Bull. 1986;17(2):22–27. 10.1111/j.1759-5436.1986.mp17002004.x

[10] Spaan E Mathijssen J Tromp N McBain F ten Have A Baltussen R. The impact of health insurance in Africa and Asia: a systematic review. Bull World Health Organ. 2012;90(9):685–692. 10.2471/BLT.12.102301. 22984313 PMC3442382

[11] Mathauer I Mathivet B Kutzin J. Community based health insurance: how can it contribute to progress towards UHC? Geneva: World Health Organization; 2017. http://www.who.int/health_financing/documents/community-based-health-insurance/en/. Accessed November 13, 2017.

[12] Rayens MK Hahn EJ. Building consensus using the policy Delphi method. Policy Polit Nurs Pract. 2000;1(4):308–315. 10.1177/152715440000100409

[13] Atun R de Andrade LOM Almeida G. Health-system reform and universal health coverage in Latin America. Lancet. 2015;385(9974):1230–1247. 10.1016/S0140-6736(14)61646-9 25458725

[14] World Health Organization (WHO). Making Fair Choices on the Path to Universal Health Coverage. Final Report of the WHO Consultative Group on Equity and Universal Health Coverage. Geneva: WHO; 2014. http://www.who.int/choice/documents/making_fair_choices/en/. Accessed November 13, 2017.

[15] McIntyre D Ranson MK Aulakh BK Honda A. Promoting universal financial protection: evidence from seven low- and middle-income countries on factors facilitating or hindering progress. Health Res Policy Syst. 2013;11(1):36. 10.1186/1478-4505-11-36 24228762 PMC3848816

[16] Rodney AM Hill PS. Achieving equity within universal health coverage: a narrative review of progress and resources for measuring success. Int J Equity Health. 2014;13(1):72. 10.1186/s12939-014-0072-8. 25928840 PMC4192297

[17] Nicholson D Yates R Warburton W Fontana G. Delivering Universal Health Coverage: A Guide for Policymakers. Report of the WISH Universal Health Coverage Forum 2015. [Doha, Qatar]: World Innovation Summit for Health (WISH); 2015. https://www.imperial.ac.uk/media/imperial-college/institute-of-global-health-innovation/public/Universal-health-coverage.pdf. Accessed November 13, 2017.

[18] Kutzin J. Anything goes on the path to universal health coverage? No. Bull World Health Organ. 2012;90(11):867–868. 10.2471/BLT.12.113654 23226900 PMC3506412

[19] Kutzin J Yip W Cashin C. Alternative financing strategies for universal health coverage. In: Scheffler RM, ed. World Scientific Handbook of Global Health Economics and Public Policy. Hackensack, NJ: World Scientific Publishing; 2016: 267–309.

[20] Jowett M Kutzin J. Raising Revenues for Health in Support of UHC: Strategic Issues for Policy Makers. Geneva: World Health Organization; 2015. http://www.who.int/health_financing/documents/revenue_raising/en/. Accessed November 13, 2017.

[21] Lagarde M Palmer N. The impact of user fees on health service utilization in low- and middle-income countries: how strong is the evidence? Bull World Health Organ. 2008;86(11):839–848. 10.2471/BLT.07.049197. 19030689 PMC2649541

[22] Ridde V Morestin F. A scoping review of the literature on the abolition of user fees in health care services in Africa. Health Policy Plan. 2011;26(1):1–11. 10.1093/heapol/czq021. 20547653

[23] Ridde V Robert E Meessen B. A literature review of the disruptive effects of user fee exemption policies on health systems. BMC Public Health. 2012;12(1):289. 10.1186/1471-2458-12-289 22521207 PMC3370991

[24] Hatt LE Makinen M Madhavan S Conlon CM. Effects of user fee exemptions on the provision and use of maternal health services: a review of literature. J Health Popul Nutr. 2013;31(4 suppl 2):67–80. 24992804 PMC4021702

[25] Richard F Antony M Witter S. Fee exemption for maternal care in sub-Saharan Africa: a review of 11 countries and lessons for the region. Glob Health Gov. September 18, 2013. https://blogs.shu.edu/ghg/2013/09/18/fee-exemption-for-maternal-care-in-sub-saharan-africa-a-review-of-11-countries-and-lessons-for-the-region/. Accessed May 3, 2108.

[26] Dzakpasu S Powell-Jackson T Campbell OMR. Impact of user fees on maternal health service utilization and related health outcomes: a systematic review. Health Policy Plan. 2014;29(2):137–150. 10.1093/heapol/czs142 23372035

[27] Crowley GR Hoffer AJ. The effects of dedicating tax revenues. Mercat Policy. June 14, 2012. https://www.mercatus.org/publication/effects-dedicating-tax-revenues. Accessed November 13, 2017.

[28] Cashin C Sparkes S Bloom D. Earmarking for Health: From Theory to Practice. Geneva: World Health Organization; 2017. http://www.who.int/health_financing/documents/earmarking-for-health/en/. Accessed November 14, 2017.

[29] Reeves A Gourtsoyannis Y Basu S McCoy D McKee M Stuckler D. Financing universal health coverage–effects of alternative tax structures on public health systems: cross-national modelling in 89 low-income and middle-income countries. Lancet. 2015;386(9990):274–280. 10.1016/S0140-6736(15)60574-8. 25982041 PMC4513966

[30] Nakhimovsky S Langenbrunner J White J Vogus A Zelelew H Avila C. Domestic Innovative Financing for Health: Learning From Country Experience. Bethesda, MD: Health Finance & Governance Project, Abt Associates Inc.; 2014. https://www.hfgproject.org/domestic-innovative-financing-health-learning-country-experience/. Accessed May 3, 2018.

[31] Cashin C. Health Financing Policy: The Macroeconomic, Fiscal, and Public Finance Context. Washington, DC: World Bank; 2016. http://documents.worldbank.org/curated/en/394031467990348481/Health-financing-policy-the-macroeconomic-fiscal-and-public-finance-context. Accessed November 14, 2017.

[32] Acharya A Vellakkal S Taylor F. The Impact of Health Insurance Schemes for the Informal Sector in Low- and Middle-Income Countries: A Systematic Review. Policy Research Working Paper No. 6324. Washington, DC: World Bank; 2013. http://documents.worldbank.org/curated/en/952181468340141917/The-impact-of-health-insurance-schemes-for-the-informal-sector-in-low-and-middle-income-countries-a-systematic-review. Accessed November 14, 2017.

[33] Nosratnejad S Rashidian A Dror DM. Systematic review of willingness to pay for health insurance in low and middle income countries. PLoS One. 2016;11(6):e0157470. 10.1371/journal.pone.0157470. 27362356 PMC4928775

[34] Van Herck P De Smedt D Annemans L Remmen R Rosenthal MB Sermeus W. Systematic review: effects, design choices, and context of pay-for-performance in health care. BMC Health Serv Res. 2010;10(1):247. 10.1186/1472-6963-10-247 20731816 PMC2936378

[35] Emmert M Eijkenaar F Kemter H Esslinger AS Schöffski O. Economic evaluation of pay-for-performance in health care: a systematic review. Eur J Health Econ. 2012;13(6):755–767. 10.1007/s10198-011-0329-8 21660562

[36] Witter S Fretheim A Kessy FL Lindahl AK. Paying for performance to improve the delivery of health interventions in low- and middle-income countries. Cochrane Database Syst Rev. 2012;(2):CD007899. 10.1002/14651858.CD007899.pub2. 22336833

[37] Eijkenaar F Emmert M Scheppach M Schöffski O. Effects of pay for performance in health care: a systematic review of systematic reviews. Health Policy. 2013;110(2-3):115–130. 10.1016/j.healthpol.2013.01.008. 23380190

[38] Das A Gopalan SS Chandramohan D. Effect of pay for performance to improve quality of maternal and child care in low- and middle-income countries: a systematic review. BMC Public Health. 2016;16(1):321. 10.1186/s12889-016-2982-4. 27074711 PMC4831162

[39] Turcotte-Tremblay AM Spagnolo J De Allegri M Ridde V. Does performance-based financing increase value for money in low- and middle- income countries? A systematic review. Health Econ Rev. 2016;6(1):30. 10.1186/s13561-016-0103-9. 27472942 PMC4967066

[40] Wiysonge CS Paulsen E Lewin S. Financial arrangements for health systems in low-income countries: an overview of systematic reviews. Cochrane Database Syst Rev. 2017;9:CD011084. 28891235 10.1002/14651858.CD011084.pub2 PMC5618470

[41] Paul E Albert L Bisala BNS . Ridde V. Performance-based financing in low-income and middle-income countries: isn't it time for a rethink? BMJ Glob Health. 2018;3:e000664. 10.1136/bmjgh-2017-000664. 29564163 PMC5859812

[42] Roehrich JK Lewis MA George G. Are public–private partnerships a healthy option? A systematic literature review. Soc Sci Med. 2014;113(suppl C):110–119. 10.1016/j.socscimed.2014.03.037. 24861412

[43] Montagu D Goodman C. Prohibit, constrain, encourage, or purchase: how should we engage with the private health-care sector? Lancet. 2016;388(10044):613–621. 10.1016/S0140-6736(16)30242-2. 27358250

[44] Pearson M Johnson M Ellison R. Review of major results based aid (RBA) and results based financing (RBF) schemes: final report. London: DFID Human Development Resource Centre; 2010. https://assets.publishing.service.gov.uk/media/57a08afb40f0b652dd000a04/Results-Based-Financing-Schemes_Report.pdf.

[45] Klingebiel S Janus H. Results-based aid: potential and limits of an innovative modality in development cooperation. Int Dev Policy. 2014;5(2). 10.4000/poldev.1746

[46] Paul E. Performance-based aid: why it will probably not meet its promises. Dev Policy Rev. 2015;33(3):313–323. 10.1111/dpr.12115

[47] Clist P. Payment by results in development aid: all that glitters is not gold. World Bank Res Obs. 2016;31(2):290–319. 10.1093/wbro/lkw005

[48] Angelsen A. REDD+ as result-based aid: general lessons and bilateral agreements of Norway. Rev Dev Econ. 2017;21(2):237–264. 10.1111/rode.12271

[49] Ekman B. Community-based health insurance in low-income countries: a systematic review of the evidence. Health Policy Plan. 2004;19(5):249–270. 10.1093/heapol/czh031 15310661

[50] Brody CM Bellows N Campbell M Potts M. The impact of vouchers on the use and quality of health care in developing countries: a systematic review. Glob Public Health. 2013;8(4):363–388. 10.1080/17441692.2012.759254 23336251

[51] Bellows NM Bellows BW Warren C. Systematic review: the use of vouchers for reproductive health services in developing countries: systematic review. Trop Med Int Health. 2011;16(1):84–96. 10.1111/j.1365-3156.2010.02667.x 21044235

[52] Lagarde M Haines A Palmer N. Conditional cash transfers for improving uptake of health interventions in low- and middle-income countries: a systematic review. JAMA. 2007;298(16):1900–1910. 10.1001/jama.298.16.1900 17954541

[53] Owusu-Addo E Renzaho AMN Smith BJ. The impact of cash transfers on social determinants of health and health inequalities in sub-Saharan Africa: a systematic review. Health Policy Plan. March 21, 2018. 10.1093/heapol/czy020 PMC5951115 29762708

[54] McIntyre D Garshong B Mtei G. Beyond fragmentation and towards universal coverage: insights from Ghana, South Africa and the United Republic of Tanzania. Bull World Health Organ. 2008;86(11):871–876. 10.2471/BLT.08.053413 19030693 PMC2649570

[55] Yip W Hafez R. Reforms for Improving the Efficiency of Health Systems: Lessons From 10 Country Cases. Synthesis Report. Geneva: World Health Organization; 2015. http://www.who.int/health_financing/documents/synthesis_report/en/. Accessed November 14, 2017.

[56] Dmytraczenko T Almeida G. Toward Universal Health Coverage and Equity in Latin America and the Caribbean: Evidence from Selected Countries. Washington, DC: World Bank; 2015. https://openknowledge.worldbank.org/handle/10986/22026. Accessed March 22, 2018.

[57] Chisholm D Evans DB. Improving Health System Efficiency as a Means of Moving Towards Universal Coverage. World Health Report (2010) Background Paper No. 28. Geneva: World Health Organization; 2010. http://www.who.int/healthsystems/topics/financing/healthreport/28UCefficiency.pdf. Accessed May 3, 2018.

[58] Siapka M Remme M Obure CD Maier CB Dehne KL Vassall A. Is there scope for cost savings and efficiency gains in HIV services? A systematic review of the evidence from low- and middle-income countries. Bull World Health Organ. 2014;92(7):499–511AD. 10.2471/BLT.13.127639. 25110375 PMC4121865

[59] Hatt LE Johns B Connor C Meline M Kukla M Moat K. Impact of Health Systems Strengthening on Health. Bethesda, MD: Health Finance & Governance Project, Abt Associates Inc.; 2015. https://www.hfgproject.org/wp-content/uploads/2016/03/Impact-of-Health-Systems-Strengthening-on-Health-7-24-1.pdf. Accessed November 14, 2017.

[60] Stigler FL Macinko J Pettigrew LM Kumar R van Weel C. No universal health coverage without primary health care. Lancet. 2016;387(10030):1811. 10.1016/S0140-6736(16)30315-4 27203497

[61] Starfield B Shi L Macinko J. Contribution of primary care to health systems and health. Milbank Q. 2005;83(3):457–502. 10.1111/j.1468-0009.2005.00409.x 16202000 PMC2690145

[62] Kringos DS Boerma WGW Hutchinson A van der Zee J Groenewegen PP. The breadth of primary care: a systematic literature review of its core dimensions. BMC Health Serv Res. 2010;10(1):65. 10.1186/1472-6963-10-65 20226084 PMC2848652

[63] Lewin S Lavis JN Oxman AD. Supporting the delivery of cost-effective interventions in primary health-care systems in low-income and middle-income countries: an overview of systematic reviews. Lancet. 2008;372(9642):928–939. 10.1016/S0140-6736(08)61403-8 18790316

[64] Kruk ME Porignon D Rockers PC Van Lerberghe W. The contribution of primary care to health and health systems in low- and middle-income countries: a critical review of major primary care initiatives. Soc Sci Med. 2010;70(6):904–911. 10.1016/j.socscimed.2009.11.025. 20089341

[65] Haas PM. Introduction: epistemic communities and international policy coordination. Int Organ. 1992;46(1):1–35. 10.1017/S0020818300001442

[66] Independent Evaluation Group. World Bank Group Support to Health Financing. Washington, DC: World Bank; 2014. https://openknowledge.worldbank.org/handle/10986/21310. Accessed November 13, 2017.

[67] Coarasa J Das J Gummerson E Bitton A. A systematic tale of two differing reviews: evaluating the evidence on public and private sector quality of primary care in low and middle income countries. Glob Health. 2017;13(1):24. 10.1186/s12992-017-0246-4. 28403871 PMC5389193

[68] Tversky A Kahneman D. Judgment under uncertainty: heuristics and biases. Science. 1974;185(4157):1124–1131. 10.1126/science.185.4157.1124 17835457

[69] Kahneman D. Thinking, Fast and Slow. New York: Farrar, Straus and Giroux; 2011.

[70] Cairney P Oliver K. Evidence-based policymaking is not like evidence-based medicine, so how far should you go to bridge the divide between evidence and policy? Health Res Policy Syst. 2017;15(1):35. 10.1186/s12961-017-0192-x 28446185 PMC5407004

[71] Wane W. The Quality of Foreign Aid: Country Selectivity or Donors Incentives? World Bank Policy Research Working Paper Series No. WPS 3325. Washington, DC: World Bank; 2004. http://documents.worldbank.org/curated/en/171951468765313837/The-quality-of-foreign-aid-country-selectivity-or-donors-incentives. Accessed November 13, 2017.

[72] Stuckler D Feigl AB Basu S McKee M. The Political Economy of Universal Health Coverage. Background paper for the Global Symposium on Health Systems Research, 16-19 November 2010, Montreux, Switzerland. Geneva: World Health Organization; 2010. https://pdfs.semanticscholar.org/3261/5063a79a268af0aeea7fbab993fb609a78b1.pdf. Accessed November 13, 2017.

[73] Savedoff WD de Ferranti D Smith AL Fan V. Political and economic aspects of the transition to universal health coverage. Lancet. 2012;380(9845):924–932. 10.1016/S0140-6736(12)61083-6 22959389

[74] O'Connell T Rasanathan K Chopra M. What does universal health coverage mean? Lancet. 2014;383(9913):277–279. 10.1016/S0140-6736(13)60955-1 23953765

[75] Baltussen R Jansen MP Mikkelsen E. Priority setting for universal health coverage: we need evidence-informed deliberative processes, not just more evidence on cost-effectiveness. Int J Health Policy Manag. 2016;5(11):615–618. 10.15171/ijhpm.2016.83 27801355 PMC5088720

[76] Gopinathan U Ottersen T. Evidence-informed deliberative processes for universal health coverage: broadening the scope. Comment on “Priority setting for universal health coverage: we need evidence-informed deliberative processes, not just more evidence on cost-effectiveness.” Int J Health Policy Manag. 2016;6(8):473–475. 10.15171/ijhpm.2016.148 PMC5553216 28812847

[77] Baltussen R Jansen MP Bijlmakers L Tromp N Yamin AE Norheim OF. Progressive realisation of universal health coverage: what are the required processes and evidence? BMJ Glob Health. 2017;2(3):e000342. 10.1136/bmjgh-2017-000342. 29082012 PMC5656135

