# Supplementary material for: Universal Health Coverage in Francophone Sub-Saharan Africa: Assessment of Global Health Experts' Confidence in Policy Options
Source: Glob Health Sci Pract. 2018 Jun 27;6(2):260–71. doi: 10.9745/GHSP-D-18-00001 (PMC6024618; doi:10.9745/GHSP-D-18-00001)
Supplement: 18-00001-Paul-FrenchSupplement.pdf [file 18-00001-Paul-FrenchSupplement.pdf]

GLOBAL HEALTH: SCIENCE AND PRACTICE
Dedicated to what works in global health programs
OPEN ACCESS

ARTICLE ORIGINAL

La couverture santé universelle en Afrique francophone au Sud du Sahara : Évaluation de la confiance d'un panel d'experts en santé mondiale quant à une série d'options stratégiques

Elisabeth Paul,[a,b] Fabienne Fecher,[a] Remo Meloni,[c] Wim van Lerberghe[d]

Même dans le contexte assez homogène de l'Afrique francophone, parmi 18 options stratégiques présentées à des experts permettant de progresser vers la couverture santé universelle (CSU), un consensus n'a été atteint que pour une option quant à son efficacité, et pour une autre quant à sa faisabilité. La complexité et les défis liés à la CSU ainsi que la faiblesse de la base de données probantes contribuent probablement à cette incertitude.

RÉSUMÉ

De nombreux pays s'appuient sur des recettes standards pour accélérer les progrès vers la couverture santé universelle (CSU). Les données empiriques généralisables étant limitées, la confiance des experts et leur consensus jouent un rôle majeur dans les choix politiques des pays. Cet article présente une tentative exploratoire menée entre avril et septembre 2016 pour mesurer la confiance et le consensus parmi un panel d'experts en santé mondiale quant à l'efficacité et la faisabilité d'un certain nombre d'options stratégiques communément proposées pour atteindre la CSU dans les pays à revenus faibles et intermédiaires, telles que les exemptions de frais pour certains groupes de personnes, les recettes affectées pour la santé et les partenariats public-privé. Pour assurer une relative homogénéité des contextes, nous nous sommes concentrés sur l'Afrique subsaharienne francophone. Nous comptions d'abord utiliser la méthode Delphi pour arriver à un consensus entre les experts, mais comme aucun consensus ne s'est profilé après deux cycles, nous avons ajusté notre approche pour passer à une analyse statistique des résultats de notre questionnaire. Nous avons mesuré le degré de consensus sur chaque option stratégique à travers 100 (signifiant le consensus total) moins l'intervalle interquartile des scores individuels attribués aux options stratégiques. Dix-sept experts en santé mondiale de divers horizons, mais ayant au moins 20 ans d'expérience dans la vaste région, ont participé aux deux cycles de l'étude. Les résultats fournissent une première « cartographie » des opinions d'un groupe d'experts et suggèrent des leçons intéressantes. Pour les 18 options stratégiques proposées, un consensus s'est seulement dégagé en faveur du renforcement de l'offre de services de soins de santé primaires de qualité (jugé efficace avec un score de confiance de 79 et un score de consensus de 90) et quant à la faisabilité des exemptions de frais pour les plus pauvres (jugées assez faciles à mettre en œuvre avec un score de confiance de 66 et un score de consensus de 85). Aucune des 18 options stratégiques communément rencontrées n'a fait l'objet d'un consensus à la fois quant à son efficacité potentielle et à sa faisabilité. Des opinions très divergentes ont été observées pour cinq options stratégiques. Le manque de confiance et de consensus au sein du groupe d'experts semble refléter le manque de preuves cohérentes sur les options proposées. Cela suggère que les opinions des experts devraient être formulées dans le cadre de processus délibératifs renforcés, inclusifs et fondés sur des données probantes, où les compromis quant aux trois dimensions de la CSU – étendre la population couverte contre les risques de santé, élargir la gamme des services et des avantages couverts, réduire les dépenses directes de santé – peuvent être discutés dans un cadre transparent et contextualisé.

INTRODUCTION

Au cours de la dernière décennie, la couverture santé universelle (CSU) est progressivement devenue l'objectif numéro un de la santé mondiale. Les progrès vers la CSU sont mesurés selon trois dimensions : (1) l'extension de la population couverte par un mécanisme de protection financière contre les risques de santé ; (2) l'élargissement de la gamme des services et prestations couverts ; et (3) la réduction des dépenses directes pour les services et prestations fournis.[1–3] Toutefois, les données probantes sur l'impact des caractéristiques et modalités spécifiques de conception de la CSU sont rares et peu concluantes,[4] et il est reconnu qu'il n'existe pas d'approche unique, standardisée pour

[a] Université de Liège, Faculté des Sciences sociales, Economie politique et économie de la santé, Liège, Belgique.
[b] Université libre de Bruxelles, Ecole de santé publique, Bruxelles, Belgique.
[c] Consultant indépendant, Kigali, Rwanda.
[d] International College of Person-Centred Medicine, Bruxelles, Belgique.
Correspondance auprès d'Elisabeth Paul (E.Paul@uliege.be).

atteindre la CSU.[2,5–8] Par conséquent, le choix entre la ou les dimensions de la CSU à privilégier en premier lieu et entre les options stratégiques à mettre en œuvre pour atteindre cet objectif implique des compromis, et exige la recherche d'un équilibre en fonction du contexte.[6] Dans la pratique, cependant, les principaux intervenants et organismes tendent souvent à éviter les décisions difficiles en s'appuyant sur des méthodes heuristiques et des politiques standardisées qui peuvent l'emporter sur la contextualisation et la négociation. L'influence des experts en santé mondiale peut être très importante à cet égard.

Cet article tente de mesurer la confiance d'experts en santé mondiale et leur consensus quant à l'efficacité et à la faisabilité d'options stratégiques couramment proposées pour atteindre la couverture santé universelle.

Cet article présente une recherche exploratoire visant à mesurer la confiance d'experts en santé mondiale quant à l'efficacité et à la faisabilité d'un certain nombre d'options stratégiques couramment proposées pour atteindre la CSU dans les pays à revenus faibles et intermédiaires (PRFI). Nous présentons d'abord nos objectifs et l'approche méthodologique que nous avons utilisée, puis nous tirons des leçons générales de nos résultats.

METHODES

Notre question de recherche découle de l'observation selon laquelle de nombreux pays s'appuient sur des recettes standards pour accélérer les progrès vers la CSU. Nous avons cherché à vérifier l'hypothèse suivante : seules les options stratégiques jugées à la fois efficaces (c'est-à-dire susceptibles d'atteindre l'objectif visé) et réalisables (c'est-à-dire raisonnablement faciles à mettre en œuvre) par une majorité d'experts devraient être mises en œuvre dans une vaste gamme de contextes ; les autres ne seraient utilisées que dans des contextes favorables et spécifiques. C'est pourquoi nous avons voulu évaluer le degré de confiance d'un échantillon d'experts en santé mondiale quant à l'efficacité potentielle et à la faisabilité d'un ensemble d'options stratégiques courantes, permettant de progresser vers la CSU dans des contextes africains typiques. Pour ce faire, nous avons opté pour la méthode Delphi dans l'espoir de parvenir à un certain degré de consensus sur au moins quelques options stratégiques sur lesquelles les pays pourraient se concentrer ou, au contraire, éviter.

Sur la base du *Rapport sur la santé dans le monde 2010* sur le financement des systèmes de santé,[2] nous avons identifié un certain nombre d'options stratégiques recommandées pour progresser vers la CSU et les avons regroupées selon les 3 dimensions du « cube » de la CSU. Nous avons sélectionné les plus fréquemment recommandées et mises en œuvre en Afrique subsaharienne francophone sur la base de notre expérience de terrain (tableau 1). Nous avons ensuite procédé à une revue sélective de la littérature sur ces politiques, à la recherche de résultats de revues systématiques si disponibles, et de données non systématiques dans le cas contraire.

Nous avons conçu un questionnaire simple comprenant des explications sur l'objectif de la recherche et deux tableaux demandant aux répondants d'évaluer le degré d'« efficacité » et de « faisabilité » potentielles des options stratégiques sur une échelle de Likert à 5 points. Pour limiter la variabilité des contextes et assurer une certaine cohérence, nous avons centré l'étude sur les contextes typiques des pays d'Afrique subsaharienne francophone ; et pour limiter les biais idéologiques, nous avons sélectionné un certain nombre d'experts en santé mondiale travaillant dans divers types d'organisations (organisations multilatérales, donateurs bilatéraux, cabinets de consultants ou consultants indépendants, universités, ministères de la santé bénéficiaires) qui avaient de l'expérience dans cette vaste région. Les experts ont été sélectionnés sur une base ad hoc pour représenter divers profils, mais 2 critères d'inclusion étaient qu'ils devaient avoir (1) au moins 20 ans d'expérience dans l'appui aux réformes de santé dans les PRFI en général, avec une expérience substantielle en Afrique francophone (occidentale et/ou centrale), et (2) un profil suffisamment généraliste pour pouvoir évaluer l'efficacité et la faisabilité des politiques relatives à la prestation de services et aux dimensions financières de la CSU.

Nous avions l'intention de mener l'enquête auprès de deux panels : l'un composé de 15 experts originaires d'Europe et d'Amérique du Nord (représentant 8 nationalités) (le panel Nord), et l'autre composé de 16 experts d'Afrique du Nord, de l'Ouest et du Centre (représentant 11 nationalités) (le panel Sud). Nous avons contacté les experts individuellement par e-mail et proposé une discussion du questionnaire par Skype s'ils le souhaitaient. Les experts n'ont pas eu accès aux cotations des autres et n'ont pas interagi pendant l'enquête. Tous les participants ont été informés des objectifs et des méthodes de l'étude et ont consenti librement à y participer. L'anonymat était garanti jusqu'à la dernière étape, lorsque nous avons partagé les résultats globaux avec les experts participants. Nous avions prévu de poursuivre le processus Delphi jusqu'à ce qu'un consensus soit atteint, puis de comparer les réponses des experts avec les données probantes disponibles sur les

TABLEAU 1. Options stratégiques courante pour tendre vers la CSU sélectionnées pour l'enquête Delphi

Dimension de la CSU	Options stratégiques
Réduction des barrières financières à l'accès	B1 Exemption de frais (gratuité sélective) pour les enfants et les femmes enceintes<br>B2 Exemption de frais (gratuité sélective) pour les plus pauvres<br>B3 Exemption de frais (gratuité sélective) pour les services prioritaires (ex. césariennes, paludisme, VIH)<br>B4 Adhésion obligatoire à un mécanisme d'assurance maladie, avec subvention des plus pauvres<br>B5 Mutuelles de santé à base communautaire<br>B6 Vouchers pour encourager l'utilisation des services de santé par les plus pauvres
Amélioration du financement des services de santé	F1 Sécurisation de ressources pour la santé à travers des crédits affectés<br>F2 Financements innovants pour la santé (ex : taxes sur le tabac (sin tax), émission de bons d'Etat, . . .)<br>F3 Mise en commun et défragmentation des mécanismes de financement existants<br>F4 Réduction des sources d'inefficience et gaspillages<br>F5 Aide liée à la performance<br>F6 Création d'un fonds mondial pour la CSU
Amélioration de l'offre et de la gestion de services	S1 Commencer par offrir un paquet limité de services à l'ensemble de la population et progressivement étendre ce paquet (approche « universaliste »)<br>S2 Commencer par offrir un paquet complet de services à certaines catégories de populations ou zones géographiques, et étendre progressivement la couverture à d'autres types de populations ou zones (approche « séquencée »)<br>S3 Commencer par renforcer l'offre de soins de santé primaires de qualité<br>S4 Renforcer le partenariat public-privé<br>S5 Paiement des prestataires et financement des services de santé sur base des résultats ou de la performance (FBR)<br>S6 Séparation des fonctions d'achat et de prestation de services à travers la création d'une agence / d'un service national(e) de santé autonome

Abréviations : B, barrières ; F, financement ; S, services ; CSU, couverture santé universelle.

options stratégiques. Les deux cycles de l'étude se sont déroulés entre avril et septembre 2016.

Adaptations apportée à la méthode Delphi

De façon inattendue, notre recherche s'est heurtée à plusieurs problèmes. Premièrement, le taux de participation a été plus faible que prévu. Sur les 31 personnes contactées, 23 ont participé au premier tour (13 du panel Nord, 10 du panel Sud) et 21 ont participé au second tour (10 Nord/ 11 Sud). Cependant, nous avons dÃ écarter quatre répondants du deuxième tour qui n'avaient pas participé au premier tour, de sorte que seuls 17 experts ont rempli les deux tours d'enquête : 9 d'Europe (représentant 5 nationalités) et 8 d'Afrique du Nord, de l'Ouest et du Centre (représentant 8 nationalités). Aucune différence substantielle n'ayant été observée entre les deux panels, nous les avons donc fusionnés en un seul panel afin d'analyser les résultats. Le tableau 2 présente les caractéristiques des 17 experts inclus dans notre panel.

Deuxièmement, après le premier tour de l'exercice, plusieurs experts ont mentionné la difficulté d'attribuer une note *in abstracto* pour chaque stratégie considérée de manière isolée. Comme il ressort de la littérature,[2,6,7] ils ont souligné la nécessité de contextualiser, d'examiner comment les stratégies interagissent les unes avec les autres et de différencier les stratégies qui peuvent être efficaces au niveau micro mais qui pourraient ne pas être facilement passées à l'échelle au niveau national (le paradoxe « micro-macro »[9]). Par exemple, bien qu'il existe des preuves solides que les mutuelles de santé communautaires améliorent l'utilisation des services et protègent financièrement leurs membres en réduisant leurs dépenses personnelles, et qu'elles améliorent la mobilisation des ressources,[10] tant la théorie que les preuves empiriques suggèrent qu'un modèle de mutuelles de santé, s'appuyant uniquement sur des programmes volontaires à petite échelle et avec une petite base de mise en commun et peu ou pas de subventions pour les groupes pauvres et vulnérables, ne peut jouer qu'un rôle très limité pour aider les pays à tendre vers la CSU.[11]

Par conséquent, à la demande des participants, nous avons converti le questionnaire initial en deux séries de deux séries de deux questions pour le deuxième tour. La première série de questions portait sur la confiance dans l'efficacité de chaque

TABLEAU 2. Caractéristiques du panel d'experts de l'étude Delphi

		Expérience dans :				
**Expert n°**	**Pays d'origine**	**Staff MS PRFI ou Assistant technique**	**Organisation multilatérale**	**Agence bilatérale**	**Agence de consultance ou consultant indépendant**	**Académique ou société civile**
1	Belgique	X	X		X	X
2	Belgique	X		X		X
3	Belgique				X	X
4	France				X	
5	Allemagne	X		X	X	
6	Allemagne	X		X		
7	Allemagne	X	X	X	X	
8	Italie		X		X	X
9	Pays-Bas	X	X		X	
10	Burundi	X			X	
11	Rép. démocratique du Congo	X	X	X	X	
12	Guinée	X	X	X	X	X
13	Côte d'Ivoire	X			X	X
14	Mali	X	X			
15	Maroc	X	X			
16	Sénégal	X			X	X
17	Tunisie	X	X			

Abréviations : PRFI, pays à revenus faibles et intermédiaires ; MS, Ministère de la Santé.

option stratégique. Ces questions ont été divisées en deux points et ont donc été notées deux fois, une fois pour leur efficacité potentielle en tant qu'intervention autonome et une fois pour leur potentiel dans le cadre d'un ensemble de réformes vers la CSU. La deuxième série de questions portait sur la confiance dans la faisabilité de chaque option politique. Ces questions ont été notées une fois pour la faisabilité opérationnelle et technique, et une fois pour la faisabilité politique. Pourtant, lorsqu'on leur a demandé de faire la distinction entre l'efficacité autonome et l'efficacité partielle, et entre la faisabilité technique et la faisabilité politique, les différences de scores étaient négligeables. Par conséquent, nous avons fusionné les scores lors de l'analyse des résultats.

Troisièmement, au cours du premier tour, les experts ont donné une réponse « modérée » à la majorité des options stratégiques, ne se positionnant donc pas clairement en faveur ou contre les options proposées. Pour permettre des réponses plus claires, nous avons converti les résultats de l'échelle de Likert en une échelle nominale de 0 à 100, et nous avons demandé aux répondants du deuxième tour d'utiliser l'échelle nominale pour se situer dans l'intervalle entre leur réponse précédente et la réponse moyenne. Cependant, au cours du deuxième tour, la plupart des options stratégiques ont continué de se situer dans la fourchette « modérément efficace », et aucun consensus ne s'est dégagé, comme nous l'espérions.

Par conséquent, nous avons changé notre approche analytique : puisqu'aucun consensus ne semblait émerger de l'utilisation de la méthode Delphi, nous avons plutôt analysé statistiquement les résultats de nos 17 questionnaires. Nous avons mesuré le degré de consensus sur chaque option stratégique par 100 moins la taille de l'écart interquartile (IQR) des scores individuels, 100 signifiant un consensus total.[12] Ceci constitue une limitation méthodologique importante de notre

TABLEAU 3. Scores moyens des participants à l'étude Delphi pour la confiance et le degré de consensus[a] quant à l'efficacité et à la faisabilité d'options stratégiques courantes vers la CSU, classées par degré croissant de confiance en l'efficacité.

		Efficacité		Faisabilité	
Dimension de la CSU	Options stratégiques	Confiance	Consensus	Confiance	Consensus
B5	Mutuelle de santé à base communautaire	40	75	49	60
F5	Aide liée à la performance	43	72	48	72,5
S5	Paiement des prestataires basé sur la performance	48	67,5	49	80
F1	Budgets affectés	48	80	56	73,75
S2	Expansion de la population couverte	49	80	54	70
F2	Financements innovants	51	80	59	65
S4	Partenariat public-privé	54	80	58	72,5
B2	Gratuité pour les plus pauvres	55	80	66	85
B6	Vouchers	56	80	63	75
F4	Réduction des sources d'inefficience	59	75	60	75
B1	Gratuité pour les enfants et femmes enceintes	60	80	64	77,5
F3	Mise en commun des mécanismes	60	75	57	65
S6	Séparation achat-prestation	60	75	60	68,75
B3	Gratuité pour des services spécifiques	61	75	65	77,5
F6	Fonds mondial pour la CSU	64	75	67	67,5
B4	Assurance santé obligatoire	67	75	68	70
S3	Renforcer l'offre de services de soins de santé primaires de qualité	79	90	78	73,75

Abréviations : B, barrières ; F, financement ; S, services ; CSU, couverture santé universelle.
[a] Degré de consensus mesuré par100 (consensus total) moins l'écart interquartile des scores individuels.

étude, en raison d'un manque de représentativité de l'échantillon d'experts. Notre panel a été initialement conçu en fonction de l'approche qualitative initiale choisie, afin de représenter un large éventail d'expériences ; cependant, il n'est pas représentatif de tous les experts en santé mondiale. Par conséquent, nos résultats quantitatifs doivent être interprétés avec prudence. Tout en donnant une indication des tendances, il ne faut certainement pas les considérer comme généralisables. D'autres limites de notre étude concernent l'accent mis sur l'Afrique francophone, qui est assez homogène à certains égards – notamment le profil épidémiologique et les principales caractéristiques des systèmes et politiques de santé – mais qui regroupe aussi, sous un même parapluie, des contextes très différents, notamment en termes de richesse et de régimes politiques. Les opinions des experts peuvent également avoir été biaisées par leur expérience personnelle, qu'elle soit positive ou négative, dans des pays spécifiques.

RESULTATS

Analyse statistique des réponses à l'enquête Delphi

En ce qui concerne l'efficacité potentielle, seules 8 options stratégiques ont fait l'objet d'un consensus de 80% ou plus, mais 7 d'entre elles se situaient dans la fourchette « modérément efficace » (scores de confiance compris entre 48% et 60%), ce qui indique un « consensus mou » (tableau 3). Seule l'option politique **« renforcer l'offre de services de soins de santé primaires de qualité »** a été jugée de manière consensuelle comme étant clairement efficace par notre panel (score de confiance de 79 avec un consensus de 90).

En ce qui concerne la faisabilité, le degré de consensus était encore plus faible. Seules deux options politiques ont reçu une note consensuelle de 80 ou plus (paiement des prestataires basé sur la performance et exemption de frais pour les plus pauvres), mais l'une d'entre elles a été jugée modérément réalisable (avec un score de

Seule l'option politique « renforcer l'offre de services de soins de santé primaires de qualité » a été jugée de manière consensuelle comme étant clairement efficace par notre panel.

confiance de 49), de sorte que seule l'option politique « exemption de frais pour les plus pauvres » a été jugée assez facile à mettre en œuvre par notre panel (score de confiance pour la faisabilité de 66, note consensuelle de 85). D'autre part, pour 5 options politiques, la note consensuelle était inférieure à 70, ce qui indique des opinions très divergentes parmi les experts.

Revue des principales données probantes sur les options stratégiques

L'expansion progressive d'un paquet de services essentiels pour l'ensemble de la population est probablement préférable à l'extension séquentielle de la part de la population couverte par un paquet complet, mais il n'existe pas de preuve systématique pour étayer cette affirmation.

Pour approfondir notre analyse, nous avons tenté de comparer les réponses de notre panel d'experts avec les données disponibles (qui ne concernent pas particulièrement l'Afrique subsaharienne francophone), afin d'évaluer si chacune correspondait ou non à l'autre. Nous avons donc choisi de rechercher des données probantes systématiques pour chacune des 18 options stratégiques proposées et nous avons complété l'analyse documentaire avec des données probantes non systématiques au besoin.

En ce qui concerne le cheminement vers la CSU, dans l'ensemble, la littérature existante conclut que l'effet des régimes de CSU sur l'accès, la protection financière et l'état de santé varie selon les contextes, la conception des régimes de CSU et les processus de mise en œuvre. Dans la plupart des pays et des régions, un certain nombre de systèmes de CSU coexistent bien qu'ils présentent une hétérogénéité en termes de conception et d'organisation. Les données probantes quant à l'impact des caractéristiques spécifiques de conception des régimes de CSU sur les résultats escomptés sont rares et peu concluantes, de sorte que plusieurs voies peuvent être appropriées.[4–7,13,14] Toutefois, il existe des indications qu'une expansion progressive d'un paquet de services essentiels pour l'ensemble de la population (parfois appelé universalisme progressif) est préférable à une expansion séquentielle de la part de la population couverte par un paquet complet de services.[14–17] Cependant, à notre connaissance, aucune preuve systématique n'est disponible pour étayer cette opinion. La littérature est également consensuelle sur le fait qu'aucun pays n'a atteint la CSU en s'appuyant principalement sur des contributions volontaires aux régimes d'assurance[18,19] et que les progrès vers la CSU requièrent l'obligation et la subvention croisée.[6,19,20]

Malgré la généralisation des partenariats public-privé, il n'existe guère de conceptualisation et de preuves empiriques approfondies sur de tels partenariats.

En ce qui concerne les options stratégiques spécifiques, nous avons constaté que le manque de confiance et de consensus parmi nos experts, avec une grande variabilité entre experts (allant de 0 à 100 dans le cas de l'exemption des frais pour les plus pauvres) et le fait que de nombreuses options stratégiques se situant dans le domaine du « consensus mou », correspond souvent au **manque de preuves cohérentes sur les options stratégiques proposées**. En effet, la littérature est souvent peu abondante, peu concluante et incohérente, ou peut remettre en question la mise en œuvre des options stratégiques proposées. C'est le cas pour :

- *Les exemptions de frais (gratuités) :* Il a été démontré qu'elles posent de nombreux problèmes de mise en œuvre, alors que les données probantes permettant de quantifier leur impact restent rares.[21–26]
- *Les ressources affectées pour la santé :* L'efficacité de l'affectation des taxes ou des recettes pour la santé semble mitigée en termes d'augmentation du financement global, d'amélioration de sa stabilité ou d'augmentation des dépenses pour le programme cible.[5,27–29]
- *Les mécanismes de financement innovants :* Ils se répartissent en différentes catégories, mais leur impact potentiel sur l'efficacité et l'équité dépend du contexte de chaque pays.[30,31]
- *L'assurance santé obligatoire :* Une revue systématique de l'impact des régimes d'assurance santé sociaux ou subventionnés par l'État n'a pas trouvé de preuves solides d'un impact sur l'utilisation, la protection contre les risques financiers ou l'état de santé.[32] De plus, la vraie question est davantage la faisabilité de la stratégie, en termes de garantie de l'adhésion à un mécanisme d'assurance maladie, parce que la volonté de payer pour l'assurance maladie dans les PRFI est faible,[33] et même les régimes subventionnés pour le secteur informel non pauvre sont confrontés à de problèmes de faibles participation et de rétention.[32]
- *Financement basé sur la performance :* Il n'existe aucune preuve cohérente de l'efficacité, de l'efficience et de l'équité de l'approche, et il y a des indications d'effets pervers possibles sur le comportement des prestataires de soins de santé et l'affaiblissement des systèmes de santé.[34–41]
- *Partenariat public-privé :* Malgré l'ampleur et l'importance du phénomène, la conceptualisation et l'étude empirique approfondie de ces partenariats sont relativement limitées. Les données probantes sur leurs effets sur la qualité clinique, la couverture, l'équité et la rentabilité sont inadéquates, et d'autres défis

concernent l'extensibilité et la portée, indiquant les limites de ces interventions comme base de la couverture santé universelle, bien que les interventions puissent aborder des problèmes ciblés sur une échelle restreinte.[42,43]

- *Séparation acheteur-prestataire :* Aucune revue systématique ou littérature cohérente n'a été trouvée sur cette question, probablement en raison de l'extrême diversité des approches.
- *Aide liée à la performance :* Ses prétendus avantages sont faussés théoriquement et mal étayés par des recherches empiriques.[44–48]

Dans l'ensemble, la qualité des preuves est faible et/ou non systématique. La confiance relative du panel d'experts dans l'assurance maladie obligatoire, la séparation acheteur-prestataire et la création d'un fonds mondial pour la CSU contraste avec le manque de preuves documentées pour ces options.

D'autre part, la littérature démontre que plusieurs options stratégiques, à l'égard desquelles nos experts avaient des opinions partagées, peuvent contribuer positivement à la CSU. C'est le cas pour :

- *Les mutuelles de santé à base communautaire :* Il existe des preuves systématiques mais faibles d'un effet modéré d'une meilleure utilisation des services et d'une protection financière à travers la réduction des dépenses directes de santé ; pourtant, ces programmes n'atteignent qu'une partie limitée de la population.[10,49]
- *Les vouchers :* Une revue systématique de l'impact des vouchers sur l'utilisation et la qualité des soins de santé dans les pays en développement a trouvé des preuves modestes que les vouchers ciblent effectivement des populations spécifiques, mais il n'y a pas suffisamment de preuves pour déterminer s'ils fournissent des soins de santé de manière efficace. Il existe des preuves solides que les vouchers augmentent l'utilisation, et des preuves modestes qu'ils améliorent la qualité,[50] en particulier pour les services de santé reproductive. Une autre revue systématique a conclu que toutes les évaluations identifiées ont fait état de certains constats positifs, indiquant que les programmes de vouchers augmentent l'utilisation des services de reproduction, améliorent la qualité des soins et améliorent les résultats en matière de santé de la population.[51] Une stratégie connexe est l'utilisation des *transferts monétaires conditionnels* : Dans l'ensemble, les données probantes suggèrent qu'ils sont efficaces pour accroître l'utilisation des services de prévention et parfois pour améliorer l'état de santé.[52] Une revue systématique récente, axée sur l'Afrique subsaharienne, a montré que les transferts monétaires peuvent être efficaces pour s'attaquer aux déterminants structurels de la santé tels que la pauvreté, l'éducation, la résilience des ménages, le travail des enfants, le capital social et la cohésion sociale. La revue a également révélé que les transferts monétaires modifient des déterminants intermédiaires tels que la nutrition, la diversité alimentaire, la privation des enfants, les comportements sexuels à risque, la grossesse chez les adolescentes et le mariage précoce. Il existe des preuves modérées selon lesquelles les transferts monétaires ont une incidence sur les impacts en termes de santé et de qualité de vie.[53]
- *La mise en commun/défragmentation des régimes :* Il existe des preuves anecdotiques d'un effet positif de cette mesure, même si l'expérience suggère qu'une fois établis, les différents régimes de fonds mis en commun sont politiquement difficiles à intégrer ou à harmoniser parce que l'intégration implique la redistribution des ressources entre les groupes d'intérêt organisés.[15,17,19,54–56]
- *La réduction des sources d'inefficience :* Il existe des preuves non systématiques d'un effet positif[57] et des preuves systématiques d'économies de coÃts et de gains d'efficacité des services liés au VIH dans les PRFI.[58]
- *Le renforcement des soins de santé primaires :* Les experts de notre panel étaient assez confiants quant au potentiel de cette option stratégique, qui est également soutenue par les bases factuelles existantes (principalement des preuves non systématiques,[5,55,59,60] mais aussi des preuves systématiques dans les pays à revenu élevé,[61,62] et des preuves systématiques limitées dans les PRFI).[63,64] Par exemple, une revue systématique a révélé que même si la majorité des programmes de soins primaires comportaient de multiples composantes – ce qui rend difficile l'attribution des effets à la seule composante des soins primaires étant donné cette intégration et la qualité variable de la recherche disponible – les initiatives de santé axées sur les soins primaires dans les PRFI ont amélioré l'accès aux soins de santé, y compris pour les pauvres, à un coÃt raisonnablement bas. Il est également prouvé que les programmes de soins primaires ont réduit la mortalité infantile et, dans certains cas, les disparités de mortalité fondées sur la richesse.[64]

Aucune des 18 options stratégiques couramment recommandées pour progresser vers la CSU n'a fait l'objet d'un consensus suffisant quant à son efficacité potentielle et à sa faisabilité.

DISCUSSION

Premièrement, comme notre groupe d'experts a été constitué sur une base *ad hoc* afin d'impliquer un large éventail d'experts généralistes et expérimentés en santé mondiale, il ne peut être caractérisé comme une communauté épistémique, c'est-à-dire « un réseau de professionnels ayant une expertise et des compétences reconnues dans un domaine particulier et pouvant prétendre à faire autorité grâce à leurs connaissances pertinentes en matière de politiques dans un domaine »[65] *(notre traduction)*. En effet, si les critères d'inclusion visaient à garantir une base de connaissances communes sur la CSU et éventuellement des croyances fondées sur des principes communs, leur hétérogénéité réelle en termes d'expérience personnelle et professionnelle – ce qui était souhaité en premier lieu – a pu conduire à des croyances causales différentes et éventuellement à des intérêts ou des valeurs idéologiques différents. Ceci a pu conduire à l'impossibilité de parvenir à un consensus par la méthode Delphi.

Nos résultats suggèrent que les options stratégiques vers la CSU ne devraient être mises en œuvre que dans des contextes favorables, après une analyse minutieuse et un processus de compromis.

Notre étude fournit une première « cartographie » des opinions d'un groupe d'experts, qui offre des leçons intéressantes. Elle montre que parmi un panel de 17 experts expérimentés en santé mondiale provenant de pays et d'horizons divers, **aucune des 18 options stratégiques couramment recommandées pour progresser vers la CSU n'a fait l'objet d'un consensus suffisant quant à son efficacité potentielle et à sa faisabilité**. Ce manque de clarté et de consensus sur les options stratégiques de la CSU dans une région relativement homogène comme l'Afrique subsaharienne francophone est en soi un élément d'information important. Selon notre hypothèse de base, cela suggère qu'**il faut être prudents lorsqu'il s'agit de mettre en œuvre ces options stratégiques et qu'elles ne devraient être mises en œuvre que dans des contextes favorables, après une analyse minutieuse et un processus de compromis**. Mais ce n'est pas le cas dans la pratique, puisque l'on observe la mise en œuvre concomitante de nombreuses options proposées dans la plupart des pays d'Afrique subsaharienne et la tendance de certains bailleurs de fonds à « vendre » des options stratégiques privilégiées quel que soit le contexte. L'exemple le plus frappant est probablement celui du financement basé sur la performance ou sur les résultats : il s'agit de l'option stratégique qui a reçu le moins de consensus parmi notre panel d'experts, ce qui contraste avec son expansion rapide financée par les bailleurs de fonds dans les PRFI. Par exemple, depuis sa création en 2007, le *Health Results Innovation Trust Fund* géré par la Banque mondiale s'est engagé à injecter 385,6 millions de dollars dans 35 programmes de financement basé sur les résultats dans 29 pays, la majeure partie des décaissements ayant eu lieu au cours des trois dernières années (https://www.rbfhealth.org/projects). Cela a été fait malgré le fait qu'en 2014, le Groupe indépendant d'évaluation de la Banque mondiale avait levé un drapeau en soulignant que la Banque avait promu le financement basé sur les résultats sans avoir un appui empirique suffisant quant à la solidité de l'approche, proclamant même que « ... des décisions ont été prises de passer à l'échelle malgré des résultats faibles, non concluants ou incomplets des projets pilotes »[66] *(notre traduction)*.

Lorsque nous avons comparé nos résultats avec les conclusions de la littérature, nous avons été frappés par l'absence de données probantes cohérentes et systématiques sur l'efficacité de la plupart des options stratégiques. De façon plus inquiétante, même les bases factuelles supposées systématiques peuvent ne pas être entièrement fiables lorsqu'elles s'appliquent à des questions complexes. Par exemple, Coarasa et al (2017) ont examiné deux revues systématiques de la littérature sur la qualité des soins primaires du secteur privé dans les PRFI, publiées dans la même revue dans un délai d'un an et aboutissant à des conclusions contradictoires. Ils concluent que la faiblesse des preuves sous-jacentes, plutôt que la rigueur des revues elles-mêmes, a conduit à des désaccords, et donc **appellent à davantage de preuves empiriques de haute qualité sur les réformes visant à atteindre la CSU**.[67] Compte tenu de l'ampleur de l'objectif de la CSU ainsi que de la complexité et de la difficulté à mettre en œuvre des réformes politiques – en particulier dans des environnements à faibles ressources comme en Afrique subsaharienne francophone – et compte tenu de l'absence de preuves solides sur les options stratégiques possibles, il peut donc être considéré très rationnel pour les experts d'avoir des opinions très différentes quant à leurs recommandations. Ceci souligne **l'importance de donner des conseils politiques et stratégiques contextualisés**.

Nous avons également été frappés par la faible différenciation de la part des experts entre, d'une part, l'efficacité des options stratégiques prises isolément et en tant que partie d'un paquet de réformes d'une part et, d'autre part, entre la faisabilité technique et la faisabilité politique (comme expliqué ci-dessus, les différences de scores étaient négligeables, de sorte que nous

avons fusionné ces deux séries de questions lors de l'analyse des résultats). Nous émettons l'hypothèse qu'il y a une disjonction entre la rationalité professionnelle des experts (ils sont intellectuellement convaincus de la nécessité de contextualiser et de différencier l'efficacité et la faisabilité dans des systèmes complexes) et une appréciation plutôt heuristique lorsqu'on leur demande de juger une stratégie. Ce phénomène est reconnu depuis longtemps[68,69] et montre la difficulté de formuler des recommandations objectives dans un système complexe. Cela peut également soulever des doutes dÃ au fait que même des experts avertis et expérimentés peuvent être soumis à des biais lors de la formulation de recommandations politiques.

Sur cette question, Cairney et Oliver (2017) identifient deux dilemmes importants pour les scientifiques et les chercheurs – que nous pouvons extrapoler aux consultants et autres conseillers – contribuant à un écart entre les politiques et les données probantes. Premièrement, dans la réalité, les acteurs combinent les données probantes avec des techniques de manipulation émotionnelle pour influencer l'agenda politique. Deuxièmement, lorsqu'ils s'adaptent à l'élaboration de politiques à plusieurs niveaux, les experts ne devraient pas nécessairement privilégier l'élaboration de politiques « fondées sur des données probantes » par rapport à d'autres principes de gouvernance tels que le processus de « coproduction » des politiques entre les organismes publics locaux, les groupes d'intérêt et les utilisateurs de services, qui peut être fondé principalement sur des valeurs. Ils concluent qu'un engagement réussi dans l'élaboration de politiques fondées sur des données probantes exige du pragmatisme, combinant les données scientifiques et les principes de gouvernance, ainsi que de la persuasion pour traduire des données probantes complexes en histoires simples. Pour maximiser l'utilisation des preuves scientifiques en matière de politiques de santé publique, les experts devraient : reconnaître la tendance des décideurs à fonder leurs jugements sur leurs croyances, leurs raccourcis émotionnels et leur connaissance de l'information ; être prêts à s'engager dans des stratégies à long terme pour pouvoir influencer les politiques ; et, dans les deux cas, décider jusqu'où ils sont prêts à aller pour persuader les décideurs d'agir et garantir une hiérarchie de preuves soutenant les politiques. Il s'agit de choix politiques et axés sur des valeurs, et pas seulement de choix « fondés sur des données probantes ».[70]

Enfin, nous nous sommes demandé pourquoi les organisations internationales continuent souvent à promouvoir un certain nombre de stratégies à la mode, dont l'efficacité manque de preuves cohérentes. Wane (2004) établit théoriquement et empiriquement que la qualité de l'aide est endogène au système d'incitation qui prévaut dans l'agence d'aide (préoccupations carriéristes) ainsi qu'à la redevabilité et à la capacité du pays bénéficiaire d'évaluer la qualité de l'aide. Il montre qu'une combinaison de facteurs (l'intérêt des gouvernements bénéficiaires d'accepter des projets s'ils apportent des avantages personnels, une culture des bailleurs de fonds de « pousser l'argent » pour augmenter les taux d'exécution, de faibles capacités et/ou une faible obligation de rendre des comptes des gouvernements bénéficiaires) conduit les gouvernements bénéficiaires à accepter des projets mal conçus au détriment de leur population.[71]

CONCLUSIONS

Malgré ses limites relevées ci-dessus, et même en limitant son focus aux contextes relativement homogènes de l'Afrique subsaharienne francophone, **cette étude exploratoire montre que les opinions d'experts en santé mondiale sur les options stratégiques permettant de progresser vers la CSU divergent beaucoup, que souvent elles ne soutiennent que modérément ou pas du tout les recettes standards qui prévalent dans la rhétorique actuelle de la santé mondiale et du développement, et que la plupart des options stratégiques ne sont pas étayées par des preuves systématiques et cohérentes**. Ceci a des implications pour la communauté du développement. Les agences de développement sont naturellement tentées de promouvoir des approches « magiques » vers la CSU. Notamment en raison d'une culture de « pousser l'argent » et des faibles capacités des gouvernements bénéficiaires,[71] les incitations tant du côté des bailleurs de fonds que du côté des bénéficiaires favorisent des solutions simples, facilement budgétisées, à instrument unique ou isolées, qui peuvent alors proliférer rapidement, même en l'absence de preuves suffisantes. Cela peut faire plus de mal que de bien.[20] L'opinion des experts a un grand rôle à jouer dans la justification des choix politiques, mais il serait dangereux de tenir leur opinion pour acquise – d'autant plus que, malgré un discours de prise de décision fondée sur des données probantes, les acteurs du développement peuvent avoir des opinions fondées principalement sur des valeurs personnelles et peuvent combiner des données probantes avec des techniques de manipulation émotionnelle

pour influencer l'agenda politique.[70] Cela fait que les conseils des experts sont politiques et fondées sur des valeurs, et pas seulement « fondés sur les données probantes ».

Par conséquent, il est clairement nécessaire de mieux comprendre les interactions entre les multiples parties prenantes – y inclus non seulement les experts mais aussi les acteurs nationaux – leurs agendas et leur hiérarchie de valeurs. Bien que la progression vers la CSU ait pris diverses formes de par le monde, une caractéristique commune qui ressort de l'expérience est que l'adoption de la CSU est avant tout une question politique plutôt que technique.[72,73] D'où la nécessité de renforcer des processus de délibération inclusifs et fondés sur les preuves permettant aux parties prenantes de discuter des compromis au regard des trois dimensions de la CSU de manière transparente, de rendre plus explicites l'ensemble des valeurs et des critères de décision utilisés, et de prendre des décisions d'une manière cohérente et contextualisée.[74–77] Cela peut aussi promouvoir plus activement le respect de l'appropriation politique nationale.

Remerciements : Nous sommes reconnaissants vis-à-vis de deux référents anonymes qui nous ont fait part de leurs commentaires sur une version antérieure de cet article.

Financement : La contribution d'EP et de FF a été soutenue par le projet « Effi-Santé » financé avec le support de la Fédération Wallonie-Bruxelles – Actions de recherche concertées – Académie Wallonie-Europe.

Intérêts concurrents : Aucun n'a été déclaré.

RÉFÉRENCES

1. World Health Organization (WHO). *The World Health Report 2008: Primary Health Care, Now More than Ever*. Geneva: WHO; 2008. http://www.who.int/whr/2008/en/. Accessed November 13, 2017.
2. World Health Organization (WHO). *The World Health Report 2010: Health Systems Financing, The Path to Universal Coverage*. Geneva: WHO; 2010. http://www.who.int/whr/2010/en/. Accessed November 13, 2017.
3. Busse R, Schreyogg J, Gericke C. *Analyzing Changes in Health Financing Arrangements in High-Income Countries: A Comprehensive Framework Approach*. HNP discussion paper. Washington, DC: World Bank; 2007. http://documents.worldbank.org/curated/en/162311468141575894/Analyzing-changes-in-health-financing-arrangements-in-high-income-countries-a-comprehensive-framework-approach. Accessed November 13, 2017.
4. Giedion U, Alfonso EA, Diaz Y. *The Impact of Universal Coverage Schemes in the Developing World: A Review of the Existing Evidence*. Universal Health Coverage (UNICO) Studies Series No. 25. Washington, DC: World Bank; 2013. http://documents.worldbank.org/curated/en/349621468158382497/The-impact-of-universal-coverage-schemes-in-the-developing-world-a-review-of-the-existing-evidence. Accessed November 13, 2017.
5. Maeda A, Cashin C, Harris J, Ikegami N, Reich MR. *Universal Health Coverage for Inclusive and Sustainable Development: A Synthesis of 11 Country Case Studies*. Washington, DC: World Bank Group; 2014. http://documents.worldbank.org/curated/en/575211468278746561/Universal-health-coverage-for-inclusive-and-sustainable-development-a-synthesis-of-11-country-case-studies. Accessed November 13, 2017.
6. Reich MR, Harris J, Ikegami N, et al. Moving towards universal health coverage: lessons from 11 country studies. *Lancet*. 2016;387(10020):811–816. CrossRef. Medline
7. Cotlear D, Nagpal S, Smith O, Tandon A, Cortez R. *Going Universal: How 24 Developing Countries Are Implementing Universal Health Coverage Reforms from the Bottom Up*. Washington, DC: World Bank; 2015. http://documents.worldbank.org/curated/en/936881467992465464/pdf/99455-PUB-Box393200B-OUO-9-PUBDATE-9-28-15-DOI-10-1596-978-1-4648-0610-0-EPI-210610.pdf. Accessed November 13, 2017.
8. World Bank. *UHC in Africa: A Framework for Action*. Washington, DC: World Bank; 2016. http://documents.worldbank.org/curated/en/735071472096342073/Main-report. Accessed November 13, 2017.
9. Mosley P. Aid-effectiveness: the micro-macro paradox. *IDS Bull*. 1986;17(2):22–27. CrossRef
10. Spaan E, Mathijssen J, Tromp N, McBain F, ten Have A, Baltussen R. The impact of health insurance in Africa and Asia: a systematic review. *Bull World Health Organ*. 2012;90(9):685–692. CrossRef. Medline
11. Mathauer I, Mathivet B, Kutzin J. Community based health insurance: how can it contribute to progress towards UHC? Geneva: World Health Organization; 2017. http://www.who.int/health_financing/documents/community-based-health-insurance/en/. Accessed November 13, 2017.
12. Rayens MK, Hahn EJ. Building consensus using the policy Delphi method. *Policy Polit Nurs Pract*. 2000;1(4):308–315. CrossRef
13. Independent Evaluation Group. *World Bank Group Support to Health Financing*. Washington, DC: World Bank; 2014. https://openknowledge.worldbank.org/handle/10986/21310. Accessed November 13, 2017.
14. Atun R, de Andrade LOM, Almeida G, et al. Health-system reform and universal health coverage in Latin America. *Lancet*. 2015;385(9974):1230–1247. CrossRef. Medline
15. World Health Organization (WHO). *Making Fair Choices on the Path to Universal Health Coverage. Final Report of the WHO Consultative Group on Equity and Universal Health Coverage*. Geneva: WHO; 2014. http://www.who.int/choice/documents/making_fair_choices/en/. Accessed November 13, 2017.
16. McIntyre D, Ranson MK, Aulakh BK, Honda A. Promoting universal financial protection: evidence from seven low- and middle-income countries on factors facilitating or hindering progress. *Health Res Policy Syst*. 2013;11(1):36. CrossRef. Medline
17. Rodney AM, Hill PS. Achieving equity within universal health coverage: a narrative review of progress and resources for measuring success. *Int J Equity Health*. 2014;13(1):72. CrossRef. Medline
18. Nicholson D, Yates R, Warburton W, Fontana G. *Delivering Universal Health Coverage: A Guide for Policymakers. Report of the WISH Universal Health Coverage Forum 2015*. [Doha, Qatar]: World Innovation Summit for Health (WISH); 2015. https://www.imperial.ac.uk/media/imperial-college/institute-of-global-health-innovation/public/Universal-health-coverage.pdf. Accessed November 13, 2017.
19. Kutzin J. Anything goes on the path to universal health coverage? No. *Bull World Health Organ*. 2012;90(11):867–868. CrossRef. Medline
20. Kutzin J, Yip W, Cashin C. Alternative financing strategies for universal health coverage. In: Scheffler RM, ed. *World Scientific Handbook of Global Health Economics and Public Policy*. Hackensack, NJ: World Scientific Publishing; 2016: 267–309.

21. Jowett M, Kutzin J. *Raising Revenues for Health in Support of UHC: Strategic Issues for Policy Makers.* Geneva: World Health Organization; 2015. http://www.who.int/health_financing/documents/revenue_raising/en/. Accessed November 13, 2017.

22. Lagarde M, Palmer N. The impact of user fees on health service utilization in low- and middle-income countries: how strong is the evidence? *Bull World Health Organ.* 2008;86(11):839–848. CrossRef. Medline

23. Ridde V, Morestin F. A scoping review of the literature on the abolition of user fees in health care services in Africa. *Health Policy Plan.* 2011;26(1):1–11. CrossRef. Medline

24. Ridde V, Robert E, Meessen B. A literature review of the disruptive effects of user fee exemption policies on health systems. *BMC Public Health.* 2012;12(1):289. CrossRef. Medline

25. Hatt LE, Makinen M, Madhavan S, Conlon CM. Effects of user fee exemptions on the provision and use of maternal health services: a review of literature. *J Health Popul Nutr.* 2013;31(4 suppl 2):67–80. Medline

26. Richard F, Antony M, Witter S, et al. Fee exemption for maternal care in sub-Saharan Africa: a review of 11 countries and lessons for the region. *Glob Health Gov.* September 18, 2013. https://blogs.shu.edu/ghg/2013/09/18/fee-exemption-for-maternal-care-in-sub-saharan-africa-a-review-of-11-countries-and-lessons-for-the-region/. Accessed May 3, 2108.

27. Dzakpasu S, Powell-Jackson T, Campbell OMR. Impact of user fees on maternal health service utilization and related health outcomes: a systematic review. *Health Policy Plan.* 2014;29(2):137–150. CrossRef. Medline

28. Crowley GR, Hoffer AJ. The effects of dedicating tax revenues. *Mercat Policy.* June 14, 2012. https://www.mercatus.org/publication/effects-dedicating-tax-revenues. Accessed November 13, 2017.

29. Cashin C, Sparkes S, Bloom D. *Earmarking for Health: From Theory to Practice.* Geneva: World Health Organization; 2017. http://www.who.int/health_financing/documents/earmarking-for-health/en/. Accessed November 14, 2017.

30. Reeves A, Gourtsoyannis Y, Basu S, McCoy D, McKee M, Stuckler D. Financing universal health coverage–effects of alternative tax structures on public health systems: cross-national modelling in 89 low-income and middle-income countries. *Lancet.* 2015;386(9990):274–280. CrossRef. Medline

31. Nakhimovsky S, Langenbrunner J, White J, Vogus A, Zelelew H, Avila C. *Domestic Innovative Financing for Health: Learning From Country Experience.* Bethesda, MD: Health Finance & Governance Project, Abt Associates Inc.; 2014. https://www.hfgproject.org/domestic-innovative-financing-health-learning-country-experience/. Accessed May 3, 2018.

32. Cashin C. *Health Financing Policy: The Macroeconomic, Fiscal, and Public Finance Context.* Washington, DC: World Bank; 2016. http://documents.worldbank.org/curated/en/394031467990348481/Health-financing-policy-the-macroeconomic-fiscal-and-public-finance-context. Accessed November 14, 2017.

33. Acharya A, Vellakkal S, Taylor F, et al. *The Impact of Health Insurance Schemes for the Informal Sector in Low- and Middle-Income Countries: A Systematic Review.* Policy Research Working Paper No. 6324. Washington, DC: World Bank; 2013. http://documents.worldbank.org/curated/en/952181468340141917/The-impact-of-health-insurance-schemes-for-the-informal-sector-in-low-and-middle-income-countries-a-systematic-review. Accessed November 14, 2017.

34. Nosratnejad S, Rashidian A, Dror DM. Systematic review of willingness to pay for health insurance in low and middle income countries. PLoS One. 2016;11(6):e0157470. CrossRef. Medline

35. Van Herck P, De Smedt D, Annemans L, Remmen R, Rosenthal MB, Sermeus W. Systematic review: effects, design choices, and context of pay-for-performance in health care. *BMC Health Serv Res.* 2010;10(1):247. CrossRef. Medline

36. Emmert M, Eijkenaar F, Kemter H, Esslinger AS, Schöffski O. Economic evaluation of pay-for-performance in health care: a systematic review. *Eur J Health Econ.* 2012;13(6):755–767. CrossRef. Medline

37. Witter S, Fretheim A, Kessy FL, Lindahl AK. Paying for performance to improve the delivery of health interventions in low- and middle-income countries. *Cochrane Database Syst Rev.* 2012;(2):CD007899. CrossRef. Medline

38. Eijkenaar F, Emmert M, Scheppach M, Schöffski O. Effects of pay for performance in health care: a systematic review of systematic reviews. *Health Policy.* 2013;110(2-3):115–130. CrossRef. Medline

39. Das A, Gopalan SS, Chandramohan D. Effect of pay for performance to improve quality of maternal and child care in low- and middle-income countries: a systematic review. *BMC Public Health.* 2016;16(1):321. CrossRef. Medline

40. Turcotte-Tremblay AM, Spagnolo J, De Allegri M, Ridde V. Does performance-based financing increase value for money in low- and middle- income countries? A systematic review. *Health Econ Rev.* 2016;6(1):30. CrossRef. Medline

41. Wiysonge CS, Paulsen E, Lewin S, et al. Financial arrangements for health systems in low-income countries: an overview of systematic reviews. *Cochrane Database Syst Rev.* 2017;9:CD011084.

42. Paul E, Albert L, Bisala BNS, et al. Ridde V. Performance-based financing in low-income and middle-income countries: isn't it time for a rethink? *BMJ Glob Health.* 2018;3:e000664. CrossRef. Medline

43. Roehrich JK, Lewis MA, George G. Are public–private partnerships a healthy option? A systematic literature review. *Soc Sci Med.* 2014;113(suppl C):110–119. CrossRef. Medline

44. Montagu D, Goodman C. Prohibit, constrain, encourage, or purchase: how should we engage with the private health-care sector? *Lancet.* 2016;388(10044):613–621. CrossRef. Medline

45. Pearson M, Johnson M, Ellison R. Review of major results based aid (RBA) and results based financing (RBF) schemes: final report. London: DFID Human Development Resource Centre; 2010. https://assets.publishing.service.gov.uk/media/57a08afb40f0b652dd000a04/Results-Based-Financing-Schemes_Report.pdf.

46. Klingebiel S, Janus H. Results-based aid: potential and limits of an innovative modality in development cooperation. *Int Dev Policy.* 2014;5(2). CrossRef

47. Paul E. Performance-based aid: why it will probably not meet its promises. *Dev Policy Rev.* 2015;33(3):313–323. CrossRef

48. Clist P. Payment by results in development aid: all that glitters is not gold. *World Bank Res Obs.* 2016;31(2):290–319. CrossRef

49. Angelsen A. REDD+ as result-based aid: general lessons and bilateral agreements of Norway. *Rev Dev Econ.* 2017;21(2):237–264. CrossRef

50. Ekman B. Community-based health insurance in low-income countries: a systematic review of the evidence. *Health Policy Plan.* 2004;19(5):249–270. CrossRef. Medline

51. Brody CM, Bellows N, Campbell M, Potts M. The impact of vouchers on the use and quality of health care in developing countries: a systematic review. *Glob Public Health.* 2013;8(4):363–388. CrossRef. Medline

52. Bellows NM, Bellows BW, Warren C. Systematic review: the use of vouchers for reproductive health services in developing countries: systematic review. *Trop Med Int Health.* 2011;16(1):84–96. CrossRef. Medline

53. Lagarde M, Haines A, Palmer N. Conditional cash transfers for improving uptake of health interventions in low- and middle-income

countries: a systematic review. *JAMA*. 2007;298(16):1900–1910. CrossRef. Medline

54. Owusu-Addo E, Renzaho AMN, Smith BJ. The impact of cash transfers on social determinants of health and health inequalities in sub-Saharan Africa: a systematic review. *Health Policy Plan*. March 21, 2018. CrossRef
55. McIntyre D, Garshong B, Mtei G, et al. Beyond fragmentation and towards universal coverage: insights from Ghana, South Africa and the United Republic of Tanzania. *Bull World Health Organ*. 2008;86(11):871–876. CrossRef. Medline
56. Yip W, Hafez R. *Reforms for Improving the Efficiency of Health Systems: Lessons From 10 Country Cases. Synthesis Report*. Geneva: World Health Organization; 2015. http://www.who.int/health_financing/documents/synthesis_report/en/. Accessed November 14, 2017.
57. Dmytraczenko T, Almeida G. *Toward Universal Health Coverage and Equity in Latin America and the Caribbean: Evidence from Selected Countries*. Washington, DC: World Bank; 2015. https://openknowledge.worldbank.org/handle/10986/22026. Accessed March 22, 2018.
58. Chisholm D, Evans DB. *Improving Health System Efficiency as a Means of Moving Towards Universal Coverage*. World Health Report (2010) Background Paper No. 28. Geneva: World Health Organization; 2010. http://www.who.int/healthsystems/topics/financing/healthreport/28UCefficiency.pdf. Accessed May 3, 2018.
59. Siapka M, Remme M, Obure CD, Maier CB, Dehne KL, Vassall A. Is there scope for cost savings and efficiency gains in HIV services? A systematic review of the evidence from low- and middle-income countries. *Bull World Health Organ*. 2014;92(7):499–511AD. CrossRef. Medline
60. Hatt LE, Johns B, Connor C, Meline M, Kukla M, Moat K. *Impact of Health Systems Strengthening on Health*. Bethesda, MD: Health Finance & Governance Project, Abt Associates Inc.; 2015. https://www.hfgproject.org/wp-content/uploads/2016/03/Impact-of-Health-Systems-Strengthening-on-Health-7-24-1.pdf. Accessed November 14, 2017.
61. Stigler FL, Macinko J, Pettigrew LM, Kumar R, van Weel C. No universal health coverage without primary health care. *Lancet*. 2016;387(10030):1811. CrossRef. Medline
62. Starfield B, Shi L, Macinko J. Contribution of primary care to health systems and health. *Milbank Q*. 2005;83(3):457–502. CrossRef. Medline
63. Kringos DS, Boerma WGW, Hutchinson A, van der Zee J, Groenewegen PP. The breadth of primary care: a systematic literature review of its core dimensions. *BMC Health Serv Res*. 2010;10(1):65. CrossRef. Medline
64. Lewin S, Lavis JN, Oxman AD, et al. Supporting the delivery of cost-effective interventions in primary health-care systems in low-income and middle-income countries: an overview of systematic reviews. *Lancet*. 2008;372(9642):928–939. CrossRef. Medline
65. Kruk ME, Porignon D, Rockers PC, Van Lerberghe W. The contribution of primary care to health and health systems in low- and middle-income countries: a critical review of major primary care initiatives. *Soc Sci Med*. 2010;70(6):904–911. CrossRef. Medline
66. Haas PM. Introduction: epistemic communities and international policy coordination. *Int Organ*. 1992;46(1):1–35. CrossRef
67. Coarasa J, Das J, Gummerson E, Bitton A. A systematic tale of two differing reviews: evaluating the evidence on public and private sector quality of primary care in low and middle income countries. *Glob Health*. 2017;13(1):24. CrossRef. Medline
68. Tversky A, Kahneman D. Judgment under uncertainty: heuristics and biases. *Science*. 1974;185(4157):1124–1131. CrossRef. Medline
69. Kahneman D. *Thinking, Fast and Slow*. New York: Farrar, Straus and Giroux; 2011.
70. Cairney P, Oliver K. Evidence-based policymaking is not like evidence-based medicine, so how far should you go to bridge the divide between evidence and policy? *Health Res Policy Syst*. 2017;15(1):35. CrossRef. Medline
71. Wane W. *The Quality of Foreign Aid: Country Selectivity or Donors Incentives?* World Bank Policy Research Working Paper Series No. WPS 3325. Washington, DC: World Bank; 2004. http://documents.worldbank.org/curated/en/171951468765313837/The-quality-of-foreign-aid-country-selectivity-or-donors-incentives. Accessed November 13, 2017.
72. Stuckler D, Feigl AB, Basu S, McKee M. *The Political Economy of Universal Health Coverage*. Background paper for the Global Symposium on Health Systems Research, 16-19 November 2010, Montreux, Switzerland. Geneva: World Health Organization; 2010. https://pdfs.semanticscholar.org/3261/5063a79a268af0aeea7fbab993fb609a78b1.pdf. Accessed November 13, 2017.
73. Savedoff WD, de Ferranti D, Smith AL, Fan V. Political and economic aspects of the transition to universal health coverage. *Lancet*. 2012;380(9845):924–932. CrossRef. Medline
74. OConnell T, Rasanathan K, Chopra M. What does universal health coverage mean? *Lancet*. 2014;383(9913):277–279. CrossRef. Medline
75. Baltussen R, Jansen MP, Mikkelsen E, et al. Priority setting for universal health coverage: we need evidence-informed deliberative processes, not just more evidence on cost-effectiveness. *Int J Health Policy Manag*. 2016;5(11):615–618. CrossRef. Medline
76. Gopinathan U, Ottersen T. Evidence-informed deliberative processes for universal health coverage: broadening the scope. Comment on "Priority setting for universal health coverage: we need evidence-informed deliberative processes, not just more evidence on cost-effectiveness." *Int J Health Policy Manag*. 2016;6(8):473–475. CrossRef. Medline
77. Baltussen R, Jansen MP, Bijlmakers L, Tromp N, Yamin AE, Norheim OF. Progressive realisation of universal health coverage: what are the required processes and evidence? *BMJ Glob Health*. 2017;2(3):e000342. CrossRef. Medline

Revu par les pairs

Reçu : décembre 20, 2017 ; **Accepté :** mars 29, 2018

© Paul et al. Ceci est un article open-access distribué sous les termes de la License Creative Commons Attribution 4.0 International (CC BY 4.0), qui permet un usage non restreint, la distribution et la reproduction sous tout medium, pourvu que les auteurs et la source originaux soient cités de façon appropriée. Pour voir une copie de la licence, visitez http://creativecommons.org/licenses/by/4.0/. Pour faire un lien vers cet article, veuillez utiliser le lien permanent suivant : https://doi.org/10.9745/GHSP-D-18-00001
